# Mental health conditions are associated with increased risk of subsequent self-harm, assault and unintentional injuries in two nations

**DOI:** 10.1038/s44220-025-00553-w

**Published:** 2025-12-22

**Authors:** Leah S. Richmond-Rakerd, Barry J. Milne, Renate M. Houts, Gabrielle Davie, Stephanie D’Souza, Sidra Goldman-Mellor, Lara Khalifeh, Avshalom Caspi, Terrie E. Moffitt, Fartein Ask Torvik

**Affiliations:** 1 Department of Psychology, University of Michigan, Ann Arbor, MI USA; 2 Centre of Methods and Policy Application in the Social Sciences, University of Auckland, Auckland, New Zealand; 3 School of Social Sciences, University of Auckland, Auckland, New Zealand; 4 Department of Psychology and Neuroscience, Duke University, Durham, NC USA; 5 Department of Preventive and Social Medicine, University of Otago, Dunedin, New Zealand; 6 Department of Public Health, School of Social Sciences, Humanities, and Arts, University of California Merced, Merced, CA USA; 7 Department of Psychiatry and Behavioral Sciences, Duke University School of Medicine, Durham, NC USA; 8 Institute of Psychiatry, Psychology, and Neuroscience, King’s College London, London, UK; 9 PROMENTA Research Center, Department of Psychology, University of Oslo, Oslo, Norway; 10 Centre for Fertility and Health, Norwegian Institute of Public Health, Oslo, Norway

**Keywords:** Risk factors, Psychiatric disorders, Epidemiology, Public health

## Abstract

Mental health conditions are associated with an increased risk of chronic physical diseases, but their implications for other physical health outcomes, including injuries, are less established. In this prospective cohort study, we tested whether mental health conditions antedate unintentional as well as self-harm and assault injuries, using administrative data from Norway (*N* = 2,753,646) and New Zealand (*N* = 2,238,813). In Norway, after accounting for pre-existing injuries, individuals with a primary care encounter for a mental health condition had an elevated risk of subsequent primary care-recorded injury. In New Zealand, as expected, individuals with a mental health-related inpatient hospital admission had an elevated risk of subsequent inpatient hospital-recorded self-harm injury, as well as assault injury. However, they also had an elevated risk of unintentional injuries. Associations extended to injury insurance claims. Associations were evident across mental health conditions, sex, age and after accounting for indicators of socioeconomic status. Risk was particularly increased for brain and head injuries. Patients with mental health conditions are an important group for injury prevention.

## Abstract (web summary)

In this two-nation administrative register study (~5 million individuals), mental health conditions were linked to subsequent unintentional, self-harm and assault injuries. These results highlight the need for targeted injury prevention strategies.

## Main

Individuals with mental health conditions are at increased risk of developing chronic age-related physical diseases^1–5^. However, the implications of mental health conditions for other physical health problems, including injuries, are less well understood. Injuries occur across the life course and account for approximately 9% of the global disease burden^6^. Despite recognition of preventative strategies—including improved safety programs and access to appropriate trauma care—injuries remain a persistent contributor to poor health and mortality^7^. Here, we provide a comprehensive analysis of the role of mental health conditions in injury risk, to broaden understanding of how mental health relates to physical well-being across the lifespan.

It is established that people with mental health conditions are more likely to experience injury from self-harm and suicide attempts^8–14^. In addition, some mental health conditions (for example, mood, psychotic and substance use disorders) are associated with a risk of injury from assaults^15–18^. Less research has considered the role of mental health conditions in the risk for unintentional injury, which is surprising as unintentional injuries constitute the majority of population injuries^19^ and account for greater disease burden and mortality than other injury types (1,338.9 disability-adjusted life-years and 22.9 deaths per 100,000 for unintentional injuries versus 877.4 disability-adjusted life-years and 16.1 deaths per 100,000 for self-harm and interpersonal violence (2019 Global Burden of Disease estimates))^7^.

Prior research on mental health conditions and unintentional injuries has mostly centered on young people, finding that children and adolescents with externalizing conditions, such as attention deficit hyperactivity disorder (ADHD) and conduct disorder, have an elevated risk^20–27^. Evidence is mixed for internalizing conditions, such as anxiety and depression, with some studies concluding that they may predispose young people to unintentional injuries^24,26,28,29^ and others concluding that they may be protective^23,30^. Few studies have assessed the associations of mental health conditions with unintentional injuries at later developmental periods^23,30,31^. With few exceptions^18,23^, most studies have relied on cross-sectional designs or follow-up periods of 5 years or less.

The current study aimed to estimate the risk of physical injuries for individuals with mental health conditions. We used population-level administrative data on nearly 5 million citizens from Norway and New Zealand aged 10–60 years at baseline, followed across periods ranging from 14 to 30 years. We tested whether mental health conditions were associated with risk for unintentional injuries, in addition to self-harm and assault injuries, because unintentional injuries constitute the bulk of population injuries. We also tested whether associations were evident across different mental health conditions to refine research into mechanisms linking mental health conditions to injuries. We tested whether associations were evident for injuries to different body systems and regions to determine the breadth of the physical health impacts of injuries. Furthermore, we tested associations in different age groups to determine whether there are sensitive periods during which mental health conditions indicate the greatest risk of injury. We also evaluated whether associations held across varying follow-up intervals to determine when the risk for injury was greatest following a mental health condition. We analyzed primary care, inpatient hospital, and injury insurance claims data to capture cases of varying severity. We controlled for pre-existing injuries to preclude reverse causation, and we controlled for indicators of socioeconomic status (educational attainment and neighborhood socioeconomic deprivation) because people’s socioeconomic status could shape or reflect common risk factors for both mental health conditions and injuries^32–34^.

## Results

### Associations of mental health conditions with injuries in Norwegian primary care data

Data about primary care contacts for the entire nation of Norway were obtained for all individuals who were born in Norway between 1946 and 1996 and resided in the country throughout the observation period extending from January 2006 through December 2019. Of the 2,753,646 individuals in the Norwegian study population, 55.7% presented to primary care for a mental health condition and 64.3% presented for an injury during the 14-year observation period.

**Fig. 1:**
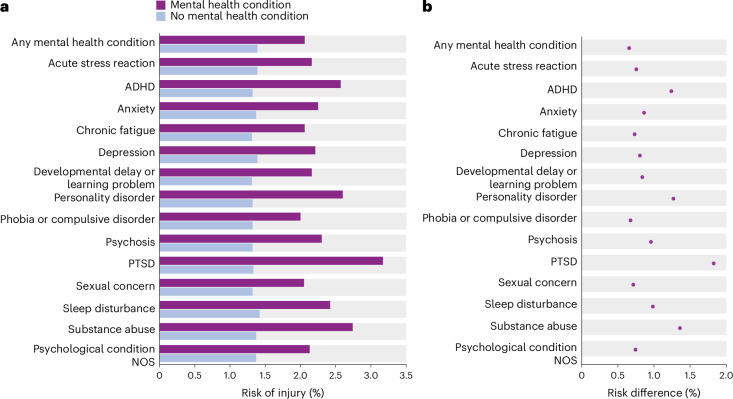
Monthly risk of primary care-recorded injuries among individuals subsequent to presenting to primary care for a mental health condition. The data are from the Norwegian study population, *N* = 2,753,646. **a**, The absolute injury risk for individuals with a mental health condition compared to individuals without a mental health condition. Estimates in **a** indicate monthly risk of injury. Inverse probability weights were used to balance mental health groups according to age at baseline, sex, county of residence and educational attainment. **b**, Differences in observed risk of injury events between the groups in **a**. The 95% CIs for risk differences in **b** are not shown as they are very narrow; the intervals are presented in Supplementary Table 1. NOS, not otherwise specified.

In each month, we determined whether individuals had a primary care encounter for a mental health condition or injury. Across 455,854,459 person-months, primary care patients with a mental health condition were more likely to subsequently experience an injury in any given month than were patients without a mental health condition, after accounting for differences in age, sex and geographical location and pre-existing injury (adjusted hazard ratio (aHR) 2.07, 95% confidence interval (CI) 2.06–2.08). Mental health conditions were linked to injuries in both sexes, ranging across age groups from an aHR of 2.11 (95% CI 2.08–2.14) to 2.25 (95% CI 2.22–2.28) in men and from an aHR of 1.86 (95% CI 1.84–1.88) to 2.57 (95% CI 2.53–2.60) in women (Table 1). Associations were reduced but remained significant after also accounting for educational attainment (Fig. 1 and Supplementary Tables 1 and 2).

**Table 1:** Associations of mental health conditions with subsequent injuries across 14 years in the Norwegian population, using diagnoses from primary care records

Birth years and age at baseline (January 2006)	Total *N*	*N* (%) men	Men			Women		
			*N* (%) with mental health condition in 14-year period^a^	*N* (%) with injury in 14-year period^b^	Adjusted HR for subsequent injury (95% CI)	*N* (%) with mental health condition in 14-year period^a^	*N* (%) with injury in 14-year period^b^	Adjusted HR for subsequent injury (95% CI)
Born 1986–1996; aged 10–19 years	550,093	285,034 (51.8)	133,441 (46.8)	204,739 (71.8)	2.25 (2.22–2.28)	160,840 (60.7)	170,920 (64.5)	2.57 (2.53–2.60)
Born 1976–1986; aged 20–29 years	474,321	244,202 (51.5)	125,548 (51.4)	163,170 (66.8)	2.19 (2.17–2.22)	157,807 (68.6)	137,171 (59.6)	2.13 (2.09–2.16)
Born 1966–1976; aged 30–39 years	591,128	301,093 (50.9)	151,513 (50.3)	196,852 (65.4)	2.12 (2.10–2.15)	196,850 (67.9)	175,291 (60.4)	1.92 (1.90–1.95)
Born 1956–1966; aged 40–49 years	576,854	292,359 (50.7)	139,851 (47.8)	187,006 (64.0)	2.14 (2.11–2.17)	183,311 (64.4)	179,170 (63.0)	1.86 (1.84–1.88)
Born 1946–1956; aged 50–60 years	561,250	283,613 (50.5)	124,236 (43.8)	177,796 (62.7)	2.11 (2.08–2.14)	159,712 (57.5)	179,465 (64.6)	1.87 (1.84–1.89)

Inverse probability weights were used to balance mental health groups according to age at baseline, sex and county of residence. Models controlled for whether individuals had an injury encounter before their encounter for a mental health condition.

^a^Mental health conditions included depression, acute stress reaction, sleep disturbance, anxiety, substance abuse, phobias or compulsive disorders, psychosis, sexual concern, ADHD, PTSD, personality disorder, developmental delays or learning problems, chronic fatigue and psychological condition not otherwise specified.

^b^Injuries included injuries in the ICPC-2 chapters: L, Musculoskeletal; S, Skin; F, Eye; N, Neurological; H, Ear; D, Digestive; and A, General and Unspecified.

**Fig. 2:**
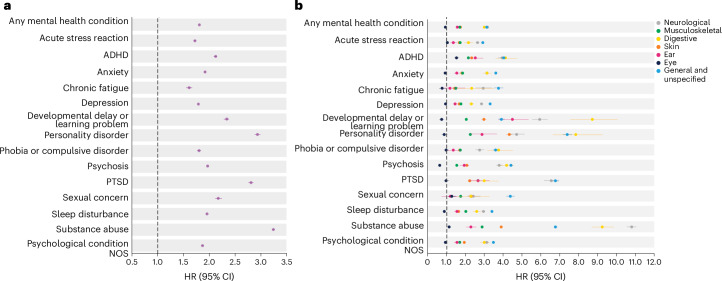
Associations of mental health conditions with risk of subsequent injuries in primary care records. The data are from the Norwegian study population, *N* = 2,753,646. **a**, HRs for associations of any mental health condition, and specific types of mental health conditions, with risk of any subsequent injury. **b**, HRs for associations of any mental health condition, and specific types of mental health conditions, with risk of subsequent injury in specific body systems. Each body system is denoted in a different color. Inverse probability weights were used to balance mental health groups according to age at baseline, sex, county of residence and educational attainment. Models controlled for whether individuals had an injury encounter before their encounter for a mental health condition. Bars indicate 95% CIs.

Every mental health condition was associated with an increased risk of subsequent injury (Fig. 2a), with the largest associations observed for substance abuse (aHR 3.25 (95% CI 3.23–3.28)), personality disorder (aHR 2.94 (95% CI 2.88–3.00)) and post-traumatic stress disorder (PTSD; aHR 2.81 (95% CI 2.76–2.86)). Pairwise analyses showed that, with the exception of injuries to the eyes and ears, all mental health conditions were associated with an increased risk of injury to all body systems (Fig. 2b). The largest associations were for injuries in the neurological (average aHR 4.16) and digestive (average aHR 4.18) systems, as well as ‘general and unspecified’ injuries, which include traumas and adverse drug effects (average aHR 4.39; Fig. 2b).

### Associations of mental health conditions with injuries in New Zealand inpatient hospital data

Data about inpatient hospital admissions for mental health conditions and injuries were obtained for all individuals who were born in New Zealand and resided in the country for any length of time during the observation period that extended from July 1989 through June 2019. Of the 2,238,813 individuals in the New Zealand Integrated Data Infrastructure (NZIDI) study population, 3.4% had an inpatient hospital admission for a mental health condition and 21.0% had an admission for an injury during the 30-year observation period. Of individuals with an injury admission, 7.3% were admitted for injury due to self-harm, 7.8% for injury due to assault and the majority (91.6%) for unintentional injuries. In addition, 0.1% and 1.8% were admitted for other and undetermined injuries, respectively (individuals could be admitted for more than one injury type). Individuals with a mental health admission constituted only 3.4% of the population but accounted for 14.2% of all injury admissions. They accounted for a disproportionate share (10.0%) even after excluding self-harm.

As expected, individuals with a mental health admission were substantially more likely to subsequently have an admission for self-harm injury (adjusted risk ratio (aRR) 25.14 (95% CI 24.38–25.91)). However, they also had over five times the risk of assault injury (aRR 5.68 (95% CI 5.47–5.90)) and over twice the risk of unintentional injury (aRR 2.17 (95% CI 2.13–2.20)), after accounting for birth year, sex and pre-existing injury. Associations were evident across age, in both men and women (Table 2). Associations were reduced but persisted after accounting for neighborhood socioeconomic deprivation (Supplementary Table 3).

**Table 2:** Associations of mental health conditions with subsequent self-harm, assault and unintentional injuries across 30 years in the New Zealand population, using diagnoses from inpatient hospital records

Men								
		*N* (%) with mental health condition or injury in 30-year period				Adjusted RR for subsequent injury (95% CI)		
**Birth years and** **age at baseline** **(July 1989)**	***N***	**Mental health condition**^**a**^	**Self-harm injury**	**Assault injury**	**Unintentional injury**	**Self-harm injury**	**Assault injury**	**Unintentional injury**
Born 1970–1979; aged 10–19 years	278,937	12,057 (4.3)	5,910 (2.1)	13,347 (4.8)	82,404 (29.5)	19.53 (18.16–21.01)	4.25 (3.97–4.55)	1.79 (1.72–1.86)
Born 1960–1969; aged 20–29 years	297,105	11,289 (3.8)	4,803 (1.6)	9,012 (3.0)	73,476 (24.7)	27.62 (25.49–29.93)	5.36 (4.97–5.79)	1.87 (1.80–1.95)
Born 1950–1959; aged 30–39 years	254,865	7,830 (3.1)	2,529 (1.0)	3,681 (1.4)	54,384 (21.3)	34.41 (30.82–38.41)	7.05 (6.25–7.95)	2.00 (1.91–2.10)
Born 1940–1949; aged 40–49 years	180,210	4,836 (2.7)	1,011 (0.6)	1,236 (0.7)	37,182 (20.6)	39.36 (32.60–47.52)	7.91 (6.29–9.96)	2.09 (1.97–2.22)
Born 1929–1939; aged 50–60 years	115,758	3,498 (3.0)	507 (0.4)	408 (0.4)	27,093 (23.4)	23.49 (17.54–31.46)	5.62 (3.47–9.09)	1.77 (1.65–1.91)

Women								
		*N* (%) with mental health condition or injury in 30-year period				Adjusted RR for subsequent injury (95% CI)		
**Birth years and** **age at baseline** **(July 1989)**	***N***	**Mental health condition**^**a**^	**Self-harm injury**	**Assault injury**	**Unintentional injury**	**Self-harm injury**	**Assault injury**	**Unintentional injury**
Born 1970–1979; aged 10–19 years	267,207	10,317 (3.9)	8,616 (3.2)	4,269 (1.6)	37,950 (14.2)	16.87 (15.81–17.99)	6.22 (5.62–6.88)	2.64 (2.52–2.77)
Born 1960–1969; aged 20–29 years	289,827	10,146 (3.5)	6,219 (2.1)	2,913 (1.0)	34,278 (11.8)	26.80 (25.03–28.71)	7.55 (6.69–8.53)	2.71 (2.59–2.84)
Born 1950–1959; aged 30–39 years	251,049	6,492 (2.6)	3,066 (1.2)	1,113 (0.4)	28,068 (11.2)	35.61 (32.27–39.29)	11.32 (9.34–13.73)	2.93 (2.77–3.10)
Born 1940–1949; aged 40–49 years	181,458	4,608 (2.5)	1,248 (0.7)	357 (0.2)	25,164 (13.9)	47.71 (40.45–56.27)	10.24 (7.01–14.96)	2.74 (2.58–2.91)
Born 1929–1939; aged 50–60 years	122,391	4,038 (3.3)	516 (0.4)	144 (0.1)	29,670 (24.2)	37.32 (28.63–48.66)	3.59 (1.55–8.35)	1.83 (1.72–1.95)

Models controlled for birth year and whether individuals had an injury admission before their first admission for a mental health condition. Per Statistics New Zealand confidentiality rules, counts were randomly rounded to base three.

^a^Mental health conditions included substance use, psychotic, mood, neurotic (anxiety), physiological disturbance, personality, developmental, behavioral and unspecified disorders.

Different types of mental health conditions were all associated with a greater risk of injuries, even after excluding self-harm from the injury outcome. The risk (aRR) of injuries ranged from 2.10 (95% CI 2.03–2.17) for psychotic disorders to 2.68 (95% CI 2.61–2.75) for substance use disorders, and applied to every age group among both men and women (Supplementary Table 4).

**Fig. 3:**
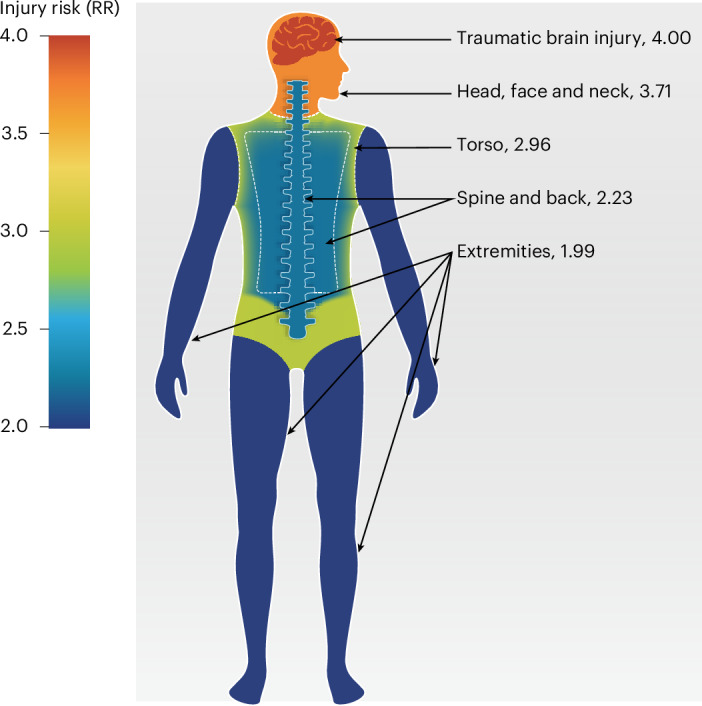
Heat map for associations of mental health conditions with subsequent injuries to different body regions in inpatient hospital records. The data are from the NZIDI study population, *N* = 2,238,813. We classified injuries according to body region using the Barell matrix^60,61^. Injuries from self-harm were excluded. Models controlled for birth year, sex and whether individuals had an injury admission before their first admission for a mental health condition. Estimates are risk ratios (RR); 95% CIs are reported in the main text.

The heat map of the human body (Fig. 3) shows that individuals with an inpatient hospital admission for a mental health condition had an increased risk of injury to every body region, even after excluding self-harm from the injury outcome. Individuals with a mental health admission had a markedly elevated risk of an admission with a primary diagnosis of traumatic brain injury (aRR 4.00 (95% CI 3.87–4.15)) and other injuries to the head, face and neck (aRR 3.71 (95% CI 3.59–3.84)), followed by an increased risk for injuries to their torso (aRR 2.96 (95% CI 2.86–3.07)), spine and back (aRR 2.23 (95% CI 2.11–2.36)) and extremities (aRR 1.99 (95% CI 1.95–2.03)). They also had an increased risk for injuries coded as unclassified according to the Barrell matrix, most of which included injuries from foreign bodies, accidental poisonings, falls and late effects of accidental injury (aRR 8.06 (95% CI 7.84–8.28)). This pattern of associations was evident across men and women of all ages (Supplementary Table 5).

The risk of injury peaked in the first year after a mental health admission (aRR 3.66 (95% CI 3.51–3.82)) but remained elevated across the follow-up period and up to 25–30 years later (aRR 2.45 (95% CI 2.30–2.61)), even after excluding self-harm from the injury outcome (Supplementary Table 6).

### Associations of mental health conditions with injuries in New Zealand injury insurance claims data

Data were also obtained about insurance claims for injuries from records maintained by the New Zealand Accident Compensation Corporation (ACC), a system that provides no-fault personal injury coverage, including coverage for treatment costs, rehabilitation costs and costs associated with income maintenance and life adaptations. Of the 2,238,813 individuals in the New Zealand study population, 2,159,814 were still alive in July 2000 when ACC records became available and resided in the country for any length of time during the 19-year ACC observation period (July 2000 through June 2019). Of these individuals, 72.5% were identified as having an injury in the ACC records during the 19-year period. After controlling for birth year, sex and pre-existing ACC-recorded injury, individuals with a mental health admission were modestly more likely to subsequently have an ACC-recorded injury (aRR 1.14 (95% CI 1.13–1.15)) and they accumulated more injuries (adjusted incidence rate ratio (aIRR) 1.35 (95% CI 1.33–1.36)). Associations were evident across most age groups, in both men and women. After accounting for neighborhood socioeconomic deprivation, associations were reduced but remained significant, with the exception of associations for any injury among the oldest cohorts (Supplementary Table 7a,b).

## Discussion

This longitudinal administrative register analysis of nearly 5 million citizens from two nations documents that individuals with mental health conditions have an elevated risk of subsequent injuries. This study had four key findings that provide insights into how mental health shapes physical well-being across the lifespan.

First, individuals with mental health conditions had an increased risk of not only self-harm injuries, but also assault and unintentional injuries, even after accounting for their injury histories. People with a mental health inpatient hospital admission were a small segment of the population but accounted for a disproportionate share of injury admissions, even after excluding self-harm. This suggests that mental health prevention may be an important component of injury prevention, including the prevention of unintentional injuries, which constitute the majority of injuries and incur disease burden and economic costs related to medical treatment and lost productivity^35–37^. As a way to gauge the size of observed associations, we note that associations of mental health conditions with hospital-recorded unintentional injury are comparable in size to associations previously identified for hospital-recorded chronic physical disease^3^. Prevention and treatment of mental health problems may be particularly consequential for unintentional injuries given their greater volume and tendency to occur across the lifespan.

Our results reinforce the importance of evaluating self-harm and suicide risk among individuals with mental health conditions and also of implementing evidence-based strategies to help protect people with mental health conditions from victimization^38,39^. Previous work has shown that individuals who experience assault injury are not only more likely to have a psychiatric history but are also more likely to subsequently receive a new psychiatric diagnosis^18^. Thus, ameliorating the risk for victimization among people with a mental health condition may also reduce their future risk for mental health problems. Our findings also suggest that mental health providers should consider delivering psychoeducation about individuals’ broader injury risk and should look for opportunities to intervene in acute-care contexts, such as during a mental health hospital admission. In addition, medical professionals should be sensitive to the potential for mental health conditions to impact patients’ recovery from injury and their ability to access and engage with treatment recommendations.

Second, associations with injuries were evident across different mental health conditions. Behavioral treatments targeting transdiagnostic risk mechanisms, such as emotion regulation-focused approaches^40^, may yield physical health benefits. However, the mechanisms linking different mental health conditions with injuries might vary. Individuals with externalizing conditions may sustain injuries owing to heightened impulsivity, interpersonal conflict and impaired risk appraisal; those with substance use disorders (for which associations with injuries were the largest in both the Norwegian and New Zealand data) may have an elevated injury risk owing to physical and psychological impairment from alcohol and drug use; and people with anxiety or depression may experience distraction while engaging in potentially hazardous activities or worry about the activities themselves, disrupting attentional control^24^. In addition, pharmacologic treatments might help to explain associations of mental health conditions with injuries. Psychiatric medications can be important in reducing both mental health problems and injuries. For instance, among children with ADHD, those who receive ADHD medication have a reduced risk of injuries compared to nontreated children^41^. However, it is possible that in some cases, side effects of medications, such as drowsiness, may increase injury risk. Future research should aim to clarify the factors that connect different mental health conditions with injuries, including the role of psychiatric medications—for instance, through pharmacoepidemiologic studies.

Third, associations were evident for injuries across body systems and regions but were strongest for head and neck injuries, including traumatic brain injury, for which individuals with a mental health condition had four times the risk in inpatient hospital data. Associations with neurological injuries were also among the largest in primary care data. Future studies should explore the mechanisms underlying head and neck injuries among individuals with mental health conditions. For instance, these injuries could result from experiences of interpersonal violence or from motor vehicle accidents, which are a major cause of traumatic brain injury and are more probable to occur among people with mental health conditions^42,43^. Our results also indicate a potential pathway through which mental health in early life could shape brain health in later life. Previous work has shown that people with mental health conditions have an elevated risk for dementia^44–48^, and traumatic brain injury has been identified as a key dementia risk factor^49^. The current report, together with these prior findings, suggests the hypothesis that brain injuries might be one mechanism connecting mental health conditions with dementia.

Fourth, associations were evident for people of all ages and across up to three decades of follow-up, suggesting that mental health conditions have implications for injury risk over the lifespan. The effects of injuries may vary across development. For instance, injuries may disrupt occupational functioning in midlife, while physical recovery may be more challenging in older adulthood. Indeed, an elevated risk for injuries was observed in body regions that confer an increased risk for frailty and falls^50^, including the extremities.

This study has strengths. First, we used whole-population data from two nations and ascertained mental health conditions and injuries using different methods, enabling replication across countries and across cases of varying severity. Second, using date-stamped medical records, we established the temporal precedence of mental health diagnoses before injuries, and we precluded reverse causation by controlling for injuries that predated individuals’ mental health conditions. Third, our study populations spanned a broad age range and were observed for up to three decades, allowing us to document associations across developmental periods and long follow-up.

This study also has limitations. First, treatment records miss cases where individuals do not seek care. Second, healthcare systems vary between nations, so our results may not generalize to other contexts. However, associations of some mental health conditions with injuries have been observed across different countries^23,24,26,27^. Third, the use of different data sources across countries allowed us to evaluate the elasticity of our findings but also meant that parallel analyses could not always be conducted. For instance, in the Norwegian primary care data, we could not differentiate injuries on the basis of intent. Fourth, in the NZIDI data, hospital admissions with an external-cause code for self-harm were classified as injuries; however, they could have had a primary diagnosis of injury or a primary mental health diagnosis with a secondary injury diagnosis^51^. Although some may view this as a misclassification issue, it reflects the nature of self-harm behavior, which involves both mental distress and physical injury. In addition, classifying self-harm events with a primary mental health diagnosis as injuries would exert a conservative effect on associations. Even in the presence of this conservative effect, the association of mental health conditions with self-harm is substantial, and we report it primarily as a benchmark against which to evaluate associations of mental health conditions with unintentional injuries. Fifth, owing to left censoring, we may not have measured all the injuries that predated individuals’ mental health conditions, particularly for those whose first mental health condition was recorded early in the observation period. This was more of a concern in New Zealand than in Norway, where the high rate of mental health and injury encounters and our assessment of encounters on a monthly level enabled more thorough capture of pre-existing injuries. Sixth, we measured educational attainment in Norway, which is only one dimension of socioeconomic status. However, low education level on its own is associated with an increased risk for a broad range of mental and physical health conditions^33^. Last, this observational study cannot confirm that mental health conditions cause an increased risk of injury, but our results identify injuries as a physical health difficulty frequently encountered by individuals with mental health conditions that warrants greater clinical attention.

### Conclusions

Patients with mental health conditions are an important group for prevention of a broad range of injuries. The current results indicate a need for increased public health surveillance of injuries among individuals with mental health conditions, including surveillance of unintentional and assault injuries in addition to self-harm injuries. Systematic collection of injury data among patients with mental health conditions—including regarding the mechanism of injury—could help to identify important contexts for injury prevention and inform the development of studies aimed at clarifying causal pathways linking mental health conditions to injuries. Such efforts will provide additional insights into opportunities to support both mental and physical health over the lifespan.

## Methods

Ethical approval was obtained from the Regional Committee for Research Ethics South East Norway (REK South East; approval no. 2018/434), the University of Auckland Human Participants Ethics Committee (ref. no. UAHPEC20738) and the Arts and Science Institutional Review Board at Duke University (approval no. 2022-0260).

### Study populations

This prospective cohort study included study populations from Norway and New Zealand. Both countries have nationwide healthcare and cradle-to-grave social welfare systems. Norway ranks higher on the United Nations’ Human Development Index^52^. Injuries account for a greater burden of disease in New Zealand than in Norway^6^. The burden of disease attributable to mental health conditions is modestly higher in New Zealand^6^.

#### Norwegian nationwide primary care register (study population 1)

The first study population was drawn from a register of primary care contacts for the entire nation of Norway. Primary care contacts are recorded using a unique personal number assigned to each citizen at birth. All residents of Norway are assigned a primary care physician (PCP). A referral from a PCP is usually required to access specialist services. Service is at no cost for juveniles and is markedly subsidized for adults. To be reimbursed for services, PCPs bill the Norwegian Health Economics Administration and indicate at least one primary diagnosis or reason for a patient’s visit. It is therefore unlikely that primary care visits go unreported^53^.

**Extended Data Fig. 1:**
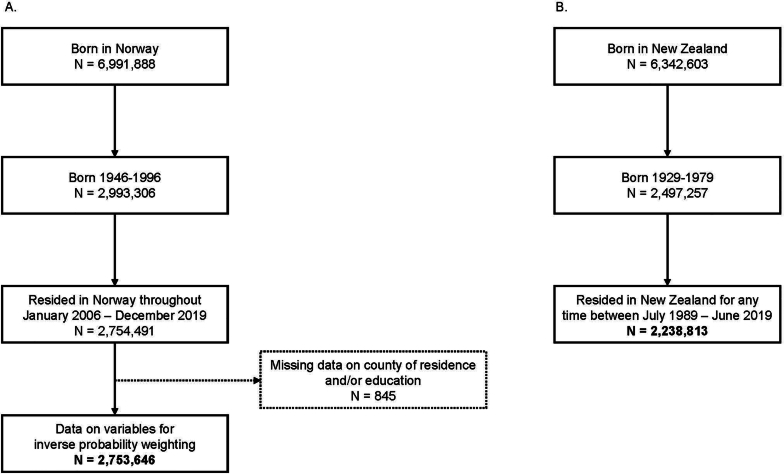
Study population selection process. This figure shows the selection processes for the Norwegian (**A**) and New Zealand (**B**) study populations.

We obtained data for all individuals who were born in Norway between 1946 and 1996, resided in Norway throughout the observation period (January 2006 through December 2019) and had information on variables used to construct inverse probability weights (*N* = 2,753,646, 51.1% male, age at baseline 10–60 years, age at the end of the 14-year observation period 24–74 years; Extended Data Fig. 1; see ‘Statistical analysis’ for more information about the inverse probability weighting). Information about ethnicity is not available in Norwegian administrative registers. Informed consent was not required for the analyses owing to the use of de-identified administrative data.

##### Mental health conditions

We obtained information about primary diagnoses of mental health conditions made in primary care^53^. Information was coded according to the International Classification of Primary Care, second edition^54^ (ICPC-2; Supplementary Methods 1). We examined 14 mental health conditions experienced by ≥1% of the population: depression, acute stress reaction, sleep disturbance, anxiety, substance abuse, phobias or compulsive disorders, psychosis, sexual concern, ADHD, PTSD, personality disorder, developmental delays or learning problems, chronic fatigue and psychological condition NOS (Supplementary Methods 2).

##### Injuries

We obtained information about primary diagnoses of injuries made in primary care. The ICPC-2 comprises chapters representing health conditions in different body systems (Supplementary Methods 3). We analyzed all injuries, and also separately, in systems where ≥1% of the population experienced an injury: chapters L, Musculoskeletal; S, Skin; F, Eye; N, Neurological; H, Ear; D, Digestive; and A, General and Unspecified. Other chapters that together constituted only 1.1% of injuries were excluded (Supplementary Methods 3). The ICPC-2 does not classify injuries by intent.

#### New Zealand Integrated Data Infrastructure (study population 2)

The second study population was drawn from the NZIDI, a collection of de-identified, individually linked, whole-of-population administrative data sources^55,56^. In the NZIDI, linkage of administrative databases at the individual level is conducted using probabilistic algorithms; false-positive linkage rates are very low^55^. We obtained data for all individuals born in New Zealand between 1929 and 1979 who resided in New Zealand for any time during the observation period extending from July 1989 through June 2019 (*N* = 2,238,813, 50.3% male, age at baseline 10–60 years, age at the end of the 30-year observation period 40–90 years; Extended Data Fig. 1). Of the study population, 1,692,612 individuals had ethnicity information, of whom 64.6% identified as European; 13.9% as Māori; 7.4% as Pacific; 10.5% as Asian; 2.4% as Middle Eastern, Latin American and African; and 1.3% as Other. Individuals could identify with more than one ethnic group. Ethnicity data are collated from multiple ranked data sources, with census data given the highest priority; individuals are assigned the ethnic profile from the highest-ranked source available for them^57^.

Output data underwent confidentiality review by Statistics New Zealand–Tatauranga Aotearoa. Informed consent was not required for analyses, following Rule 11(2)(c)(iii) of the New Zealand Health Information Privacy Code^58^. Under certain circumstances, this rule allows for anonymized health data to be used for research without the authorization of the individual concerned.

##### Mental health conditions

We obtained information about publicly funded mental health admissions to inpatient hospitals from records maintained by the New Zealand Ministry of Health. Most of New Zealand’s medical treatment is provided in public hospitals^59^. Acute and nonacute services (including medical, emergency, diagnostic, surgical and maternity services) are provided for both mental and physical health concerns. Public hospital records include almost all hospitalizations; only about 5% of New Zealand’s hospitalizations occur in private hospitals, with most being for elective surgical procedures^59^.

Information was coded according to the International Classification of Diseases, ninth and tenth revisions (ICD-9 and ICD-10). Using primary diagnoses, we classified nine categories of mental health conditions, termed ‘disorders’ in the ICD: substance use, psychotic, mood, neurotic (anxiety), physiological disturbance, personality, developmental, behavioral and unspecified disorders^3,44^ (Supplementary Methods 4).

##### Injuries

We obtained information about publicly funded injury-related admissions to inpatient hospitals. These include direct admissions and emergency department presentations that involve treatment for more than 3 hours or result in death. We obtained primary diagnoses and external-cause codes, based on the ICD-9 and ICD-10. We classified injuries by body region using the Barell Injury Diagnosis Matrix^60,61^ (head and neck, spine and back, torso, extremities, and unclassified). We classified injury intent using the National Center for Injury Prevention and Control external cause of injury matrix^61^ (self-harm, assault, unintentional, other, and undetermined intent (Supplementary Methods 5)).

We obtained information about insurance claims for injuries from records maintained by the ACC, a system that provides no-fault personal injury coverage, including coverage for treatment costs, rehabilitation costs and costs associated with income maintenance and life adaptations. All New Zealanders who experience an injury are eligible for coverage, regardless of how the injury occurred and the setting where it was treated. Whereas inpatient hospital records were available for the 30-year observation period, ACC records were available for 19 years, from July 2000 onward (Supplementary Methods 6).

### Statistical analysis

#### Norwegian study population

As individuals often had multiple primary care encounters, our analysis accounted for event recurrence and timing. We used extended Cox proportional hazards models to test whether a mental health condition increased the risk of subsequent injury across the 455,854,459 person-months in the 14-year observation period. In each month, we determined whether individuals had an encounter for a mental health condition or injury. Independent exposures (mental health conditions) were treated as time-varying covariates. Dependent time-to-injury variables were considered recurrent events. Models were estimated using the counting-process (start, stop) approach^62^. Recurrent events were treated as identical (that is, subsequent injuries were not assumed to be more or less severe than the first injury), and time-varying exposures were parameterized to represent the overall effect on survival time^63^. The cause-specific hazard of injury was calculated, with death modeled as a competing risk. We analyzed associations for all mental health conditions together and separately by condition. Likewise, we analyzed associations for all injuries together and separately by body system. Models controlled for whether individuals had an injury encounter before their encounter for a mental health condition, to preclude reverse causation (the potential for injuries to lead to mental health problems, rather than vice versa).

We used inverse probability weighting to balance groups according to three variables: baseline age, sex assigned at birth and county of residence^5^. We accounted for county of residence because the density of primary care practices varies by residential location. We tested whether associations held after further balancing groups on educational attainment (an indicator of socioeconomic status). Analyses were performed using SAS version 9.4 TS Level 1M6 and R version 4.2.3 x64.

#### NZIDI

We first analyzed inpatient hospital records of mental health conditions and injuries. Individuals’ first admission for a mental health condition during the observation period was their index condition; those with no mental health admissions were controls. Inpatient hospital admissions are comparatively rare relative to primary care encounters. Therefore, our analysis estimated the relative risk (RR) of experiencing any subsequent injury among those with versus without a mental health condition. We used Poisson regression models to estimate RRs. We analyzed associations for all conditions together and separately for substance use, psychotic, mood and neurotic disorders. We analyzed associations for all injuries together and separately for (1) unintentional, self-harm and assault injuries and (2) injuries to different body regions. Models controlled for whether individuals had an injury admission before their index mental health admission.

As a robustness check, we tested whether associations extended to injury insurance claims. We used Poisson regression models to estimate the RRs for subsequent ACC-recorded injury. Given the high rate of ACC-recorded injuries, we also used negative binomial regression models to estimate incidence rate ratios for the number of injuries. Models controlled for whether individuals had a previous ACC-recorded injury.

Our analysis needed to account for the different duration of observation time among patients with mental health conditions, who were observed from their first mental health admission, and individuals without mental health conditions, whose observation time extended over the entire 30 years. To do this, we randomly assigned observation periods to controls to match observation durations among cases, on the basis of the distributions of admission dates for cases’ mental health admissions^3,44^ (Supplementary Methods 7). We weighted the data on the basis of time alive and residency in the country, to account for remaining differences between individuals in their observation time^3,44^. Individuals who had an injury that was ultimately fatal were identified as having had an injury; their time alive was accounted for in the weighting.

All models controlled for birth year; models using the total population additionally controlled for sex. We tested whether associations held after further controlling for neighborhood socioeconomic deprivation (an indicator of socioeconomic status)^64^. We used neighborhood deprivation rather than educational attainment because there was more missing data regarding education (owing to dates of availability for official education records and census records).

Per Statistics New Zealand confidentiality rules, reported frequencies and counts were randomly rounded to base three. Analyses were performed using SAS version 9.4.

### Statistics New Zealand disclaimer

These results are not official statistics. They have been created for research purposes from the Integrated Data Infrastructure (IDI), which is carefully managed by Statistics New Zealand. Statistics New Zealand approved the use of the IDI for this project (ref. no. 023377, MAA2019-35). For more information about the IDI, please visit https://www.stats.govt.nz/integrated-data/.

### Reporting summary

Further information on research design is available in the Nature Portfolio Reporting Summary linked to this article.

## Supplementary information

Supplementary Information Supplementary Methods 1–7, Tables 1–7 and references.

Reporting Summary

## Data Availability

The data for this study are primary care records of entire cohorts of the Norwegian population and nationwide register data from the NZIDI. The data cannot be shared by the authors because access to the data is regulated via managed-access programs. Researchers can access the Norwegian primary care data by application to the Regional Committees for Medical and Health Research Ethics and the data owners (Statistics Norway and the Norwegian Directorate of Health). Researchers who wish to use the NZIDI data must submit an application through Statistics New Zealand. Researchers can contact this report’s authors if they have questions concerning the data.

## Code availability

Statistical code is available via GitHub at https://github.com/leahrr-umich/MentalHealth-Injury.git.
