# Supplementary material for: Mental health conditions are associated with increased risk of subsequent self-harm, assault and unintentional injuries in two nations
Source: Nat Ment Health. 2025 Dec 22;4(1):102–11. doi: 10.1038/s44220-025-00553-w (PMC12789009; doi:10.1038/s44220-025-00553-w)
Supplement: Supplementary file 1 — Supplementary Methods 1–7, Tables 1–7 and references. [file 44220_2025_553_MOESM1_ESM.pdf]

nature mental health

Supplementary information https://doi.org/10.1038/s44220-025-00553-w

Mental health conditions are associated with increased risk of subsequent self-harm, assault and unintentional injuries in two nations

In the format provided by the authors and unedited

Supplementary Materials for:

Nationwide evidence that mental health conditions are associated with increased risk of subsequent self-harm, assault and unintentional injuries

Supplementary Methods 1. International Classification of Primary Care, 2nd edition (ICPC-2).
Supplementary Methods 2. ICPC-2 codes and prevalence estimates for mental-health conditions in the Norwegian study population (N=2,753,646).
Supplementary Methods 3. ICPC-2 codes and prevalence estimates for injuries in the Norwegian study population (N=2,753,646).
Supplementary Methods 4. Ascertainment of mental-health conditions in the NZIDI study population (N=2,238,813), using publicly funded inpatient-hospital records.
Supplementary Methods 5. Ascertainment of injuries in the NZIDI study population (N=2,238,813), using publicly funded inpatient-hospital records.
Supplementary Methods 6. Ascertainment of injuries in the NZIDI study population (N=2,159,814), using Accident Compensation Corporation records.
Supplementary Methods 7. Random-matching procedure in the NZIDI study population.
Supplementary Table 1. Risk differences [and 95% confidence intervals] for monthly risk of injuries as a function of presenting to primary care for a mental-health condition, in the Norwegian study population.
Supplementary Table 2. Adjusted hazard ratios [and 95% confidence intervals] for associations of mental-health conditions with subsequent injuries in Norwegian primary-care records, before and after accounting for education.
Supplementary Table 3. Adjusted risk ratios [and 95% confidence intervals] for associations of mental-health conditions with subsequent self-harm, assault, and unintentional injuries in New Zealand inpatient-hospital records, before and after accounting for neighborhood socioeconomic deprivation.
Supplementary Table 4. Adjusted risk ratios [and 95% confidence intervals] for associations of mental-health conditions with subsequent injuries in the NZIDI study population, by type of mental-health condition.
Supplementary Table 5. Adjusted risk ratios [and 95% confidence intervals] for associations of mental-health conditions with subsequent injuries to different body regions, in the NZIDI study population.
Supplementary Table 6. Adjusted risk ratios for associations of mental-health conditions with subsequent injuries across varying follow-up periods, in the NZIDI study population.
Supplementary Table 7. Adjusted risk ratios and incidence rate ratios for associations of mental-health conditions with subsequent injuries, with injuries ascertained from ACC claims data, in the NZIDI study population.
Supplementary References.

Supplementary Methods 1. International Classification of Primary Care, 2nd edition (ICPC-2).

ICPC-2 – English

International Classification of Primary Care – 2nd Edition

Wonca International Classification Committee (WICC)

Wonca

Process codes

-30 Medical Exam/Eval-Complete
-31 Medical Examination/Health Evaluation-Partial/Pre-op check
-32 Sensitivity Test
-33 Microbiological/Immunological Test
-34 Blood Test
-35 Urine Test
-36 Faeces Test
-37 Histological/Exfoliative Cytology
-38 Other Laboratory Test NEC
-39 Physical Function Test
-40 Diagnostic Endoscopy
-41 Diagnostic Radiology/Imaging
-42 Electrical Tracings
-43 Other Diagnostic Procedures
-44 Preventive Imunisations/Medications
-45 Observe/Educate/Advice/Diet
-46 Consult with Primary Care Provider
-47 Consultation with Specialist
-48 Clarification/Discuss Patient's RFE
-49 Other Preventive Procedures
-50 Medicat-Script/Reqst/Renew/Inject
-51 Incise/Drain/Flush/Aspirate
-52 Excise/Remove/Biopsy/Destruction/Debride
-53 Instrument/Catheter/Intubate/Dilate
-54 Repair/Fixate-Suture/Cast/Prosthetic
-55 Local Injection/Infiltration
-56 Dress/Press/Compress/Tamponade
-57 Physical Medicine/Rehabilitation
-58 Therapeutic Counselling/Listening
-59 Other Therapeutic Procedure NEC
-60 Results Tests/Procedures
-61 Results Exam/Test/Record
-62 Administrative Procedure
-63 Follow-up Encounter Unspecified
-64 Encounter Initiated by Provider
-65 Encounter Initiated third person
-66 Refer to Other Provider (EXCL. M.D.)
-67 Referral to Physician/Specialist/Clinic/Hospital
-68 Other Referrals NEC
-69 Other Reason for Encounter NEC

General and Unspecified A

A01 Pain general/multiple sites
A02 Chills
A03 Fever
A04 Weakness/tiredness general
A05 Feeling ill
A06 Fainting/syncope
A07 Coma
A08 Swelling
A09 Sweating problem
A10 Bleeding/haemorrhage NOS
A11 Chest pain NOS
A13 Concern/fear medical treatment
A16 Irritable infant
A18 Concern about appearance
A20 Euthanasia request/discussion
A21 Risk factor for malignancy
A23 Risk factor NOS
A25 Fear of death/dying
A26 Fear of cancer NOS
A27 Fear of other disease NOS
A28 Limited function/disability NOS
A29 General symptom/complaint other
A70 Tuberculosis
A71 Measles
A72 Chickenpox
A73 Malaria
A74 Rubella
A75 Infectious mononucleosis
A76 Viral exanthem other
A77 Viral disease other/NOS
A78 Infectious disease other/NOS
A79 Malignancy NOS
A80 Trauma/injury NOS
A81 Multiple trauma/injuries
A82 Secondary effect of trauma
A84 Poisoning by medical agent
A85 Adverse effect medical agent
A86 Toxic effect non-medicinal substance
A87 Complication of medical treatment
A88 Adverse effect physical factor
A89 Effect prosthetic device
A90 Congenital anomaly OS/multiple
A91 Abnormal result investigation NOS
A92 Allergy/allergic reaction NOS
A93 Premature newborn
A94 Perinatal morbidity other
A95 Perinatal mortality
A96 Death
A97 No disease
A98 Health maintenance/prevention
A99 General disease NOS

Blood, Blood Forming Organs and Immune Mechanism B

B02 Lymph gland(s) enlarged/painful
B04 Blood symptom/complaint
B25 Fear of aids/HIV
B26 Fear cancer blood/lymph
B27 Fear blood/lymph disease other
B28 Limited function/disability
B29 Sympt/complt lymph/immune other
B70 Lymphadenitis acute
B71 Lymphadenitis non-specific
B72 Hodgkin's disease/lymphoma
B73 Leukaemia
B74 Malignant neoplasm blood other
B75 Benign/unspecified neoplasm blood
B76 Ruptured spleen traumatic
B77 Injury blood/lymph/spleen other
B78 Hereditary haemolytic anaemia
B79 Congen.anom. blood/lymph other
B80 Iron deficiency anaemia
B81 Anaemia, Vitamin B12/folate def.
B82 Anaemia other/unspecified
B83 Purpura/coagulation defect
B84 Unexplained abnormal white cells
B87 Splenomegaly
B90 HIV-infection/aids
B99 Blood/lymph/spleen disease other

PROCESS CODES
SYMPTOMS/COMPLAINTS
INFECTIONS
NEOPLASMS
INJURIES
CONGENITAL ANOMALIES
OTHER DIAGNOSES

Digestive D

D01 Abdominal pain/cramps general
D02 Abdominal pain epigastric
D03 Heartburn
D04 Rectal/anal pain
D05 Perianal itching
D06 Abdominal pain localized other
D07 Dyspepsia/indigestion
D08 Flatulence/gas/belching
D09 Nausea
D10 Vomiting
D11 Diarrhoea
D12 Constipation
D13 Jaundice
D14 Haematemesis/vomiting blood
D15 Melaena
D16 Rectal bleeding
D17 Incontinence of bowel
D18 Change faeces/bowel movements
D19 Teeth/gum symptom/complaint
D20 Mouth/tongue/lip symptom/compl.
D21 Swallowing problem
D23 Hepatomegaly
D24 Abdominal mass NOS
D25 Abdominal distension
D26 Fear of cancer of digestive system
D27 Fear of digestive disease other
D28 Limited function/disability (d)
D29 Digestive symptom/complaint other
D70 Gastrointestinal infection
D71 Mumps
D72 Viral hepatitis
D73 Gastroenteritis presumed infection
D74 Malignant neoplasm stomach
D75 Malignant neoplasm colon/rectum
D76 Malignant neoplasm pancreas
D77 Malig. neoplasm digest other/NOS
D78 Neoplasm digest benign/uncertain
D79 Foreign body digestive system
D80 Injury digestive system other
D81 Congen. anomaly digestive system
D82 Teeth/gum disease
D83 Mouth/tongue/lip disease
D84 Oesophagus disease
D85 Duodenal ulcer
D86 Peptic ulcer other
D87 Stomach function disorder
D88 Appendicitis
D89 Inguinal hernia
D90 Hiatus hernia
D91 Abdominal hernia other
D92 Diverticular disease
D93 Irritable bowel syndrome
D94 Chronic enteritis/ulcerative colitis
D95 Anal fissure/perianal abscess
D96 Worms/other parasites
D97 Liver disease NOS
D98 Cholecystitis/cholelithiasis
D99 Disease digestive system, other

Eye F

F01 Eye pain
F02 Red eye
F03 Eye discharge
F04 Visual floaters/spots
F05 Visual disturbance other
F13 Eye sensation abnormal
F14 Eye movements abnormal
F15 Eye appearance abnormal
F16 Eyelid symptom/complaint
F17 Glasses symptom/complaint
F18 Contact lens symptom/complaint
F27 Fear of eye disease
F28 Limited function/disability (f)
F29 Eye symptom/complaint other
F70 Conjunctivitis infectious
F71 Conjunctivitis allergic
F72 Blepharitis/stye/chalazion
F73 Eye infection/inflammation other
F74 Neoplasm of eye/adnexa
F75 Contusion/haemorrhage eye
F76 Foreign body in eye
F79 Injury eye other
F80 Blocked lacrimal duct of infant
F81 Congenital anomaly eye other
F82 Detached retina
F83 Retinopathy
F84 Macular degeneration
F85 Corneal ulcer
F86 Trachoma
F91 Refractive error
F92 Cataract
F93 Glaucoma
F94 Blindness
F95 Strabismus
F99 Eye/adnexa disease, other

Ear H

H01 Ear pain/earache
H02 Hearing complaint
H03 Tinnitus, ringing/buzzing ear
H04 Ear discharge
H05 Bleeding ear
H13 Plugged feeling ear
H15 Concern with appearance of ears
H27 Fear of ear disease
H28 Limited function/disability ear
H29 Ear symptom/complaint other
H70 Otitis externa
H71 Acute otitis media/myringitis
H72 Serous otitis media
H73 Eustachian salpingitis
H74 Chronic otitis media
H75 Neoplasm of ear
H76 Foreign body in ear
H77 Perforation ear drum
H78 Superficial injury of ear
H79 Ear injury other
H80 Congenital anomaly of ear
H81 Excessive ear wax
H82 Vertiginous syndrome
H83 Otosclerosis
H84 Presbyacusis
H85 Acoustic trauma
H86 Deafness
H99 Ear/mastoid disease, other

Cardiovascular K

K01 Heart pain
K02 Pressure/tightness of heart
K03 Cardiovascular pain NOS
K04 Palpitations/awareness of heart
K05 Irregular heartbeat other
K06 Prominent veins
K07 Swollen ankles/oedema
K22 Risk factor cardiovascular disease
K24 Fear of heart disease
K25 Fear of hypertension
K27 Fear cardiovascular disease other
K28 Limited function/disability (k)
K29 Cardiovascular sympt./complt. other
K70 Infection of circulatory system
K71 Rheumatic fever/heart disease
K72 Neoplasm cardiovascular
K73 Congenital anomaly cardiovascular
K74 Ischaemic heart disease w. angina
K75 Acute myocardial infarction
K76 Ischaemic heart disease w/o angina
K77 Heart failure
K78 Atrial fibrillation/flutter
K79 Paroxysmal tachycardia
K80 Cardiac arrhythmia NOS
K81 Heart/arterial murmur NOS
K82 Pulmonary heart disease
K83 Heart valve disease NOS
K84 Heart disease other
K85 Elevated blood pressure
K86 Hypertension uncomplicated
K87 Hypertension complicated
K88 Postural hypotension
K89 Transient cerebral ischaemia
K90 Stroke/cerebrovascular accident
K91 Cerebrovascular disease
K92 Atherosclerosis/PVD
K93 Pulmonary embolism
K94 Phlebitis/thrombophlebitis
K95 Varicose veins of leg
K96 Haemorrhoids
K99 Cardiovascular disease other

Musculoskeletal L

L01 Neck symptom/complain
L02 Back symptom/complaint
L03 Low back symptom/complaint
L04 Chest symptom/complaint
L05 Flank/axilla symptom/complaint
L07 Jaw symptom/complaint
L08 Shoulder symptom/complaint
L09 Arm symptom/complaint
L10 Elbow symptom/complaint
L11 Wrist symptom/complaint
L12 Hand/finger symptom/complaint
L13 Hip symptom/complaint
L14 Leg/thigh symptom/complaint
L15 Knee symptom/complaint
L16 Ankle symptom/complaint
L17 Foot/toe symptom/complaint
L18 Muscle pain
L19 Muscle symptom/complaint NOS
L20 Joint symptom/complaint NOS
L26 Fear of cancer musculoskeletal
L27 Fear musculoskeletal disease other
L28 Limited function/disability (l)
L29 Sympt/complt. Musculoskeletal other
L70 Infections musculoskeletal system
L71 Malignant neoplasm musculoskeletal
L72 Fracture: radius/ulna
L73 Fracture: tibia/fibula
L74 Fracture: hand/foot bone
L75 Fracture: femur
L76 Fracture: other
L77 Sprain/strain of ankle
L78 Sprain/strain of knee
L79 Sprain/strain of joint NOS
L80 Dislocation/subluxation
L81 Injury musculoskeletal NOS
L82 Congenital anomaly musculoskeletal
L83 Neck syndrome
L84 Back syndrome w/o radiating pain
L85 Acquired deformity of spine
L86 Back syndrome with radiating pain
L87 Bursitis/tendinitis/synovitis NOS
L88 Rheumatoid/seropositive arthritis
L89 Osteoarthrosis of hip
L90 Osteoarthrosis of knee
L91 Osteoarthrosis other
L92 Shoulder syndrome
L93 Tennis elbow
L94 Osteochondrosis
L95 Osteoporosis
L96 Acute internal damage knee
L97 Neoplasm benign/unspec musculo.
L98 Acquired deformity of limb
L99 Musculoskeletal disease, other

Neurological N

N01 Headache
N03 Pain face
N04 Restless legs
N05 Tingling fingers/feet/toes
N06 Sensation disturbance other
N07 Convulsion/seizure
N08 Abnormal involuntary movements
N16 Disturbance of smell/taste
N17 Vertigo/dizziness
N18 Paralysis/weakness
N19 Speech disorder
N26 Fear cancer neurological system
N27 Fear of neurological disease other
N28 Limited function/disability (n)
N29 Neurological symptom/complt. other
N70 Poliomyelitis
N71 Meningitis/encephalitis
N72 Tetanus
N73 Neurological infection other
N74 Malignant neoplasm nervous system
N75 Benign neoplasm nervous system
N76 Neoplasm nervous system unspec.
N79 Concussion
N80 Head injury other
N81 Injury nervous system other
N85 Congenital anomaly neurological
N86 Multiple sclerosis
N87 Parkinsonism
N88 Epilepsy
N89 Migraine
N90 Cluster headache
N91 Facial paralysis/bell's palsy
N92 Trigeminal neuralgia
N93 Carpal tunnel syndrome
N94 Peripheral neuritis/neuropathy
N95 Tension headache
N99 Neurological disease, other

Psychological P

- P01 Feeling anxious/nervous/tense
- P02 Acute stress reaction
- P03 Feeling depressed
- P04 Feeling/behaving irritable/angry
- P05 Senility, feeling/behaving old
- P06 Sleep disturbance
- P07 Sexual desire reduced
- P08 Sexual fulfilment reduced
- P09 Sexual preference concern
- P10 Stammering/stuttering/tic
- P11 Eating problem in child
- P12 Bedwetting/enuresis
- P13 Encopresis/bowel training problem
- P15 Chronic alcohol abuse
- P16 Acute alcohol abuse
- P17 Tobacco abuse
- P18 Medication abuse
- P19 Drug abuse
- P20 Memory disturbance
- P22 Child behaviour symptom/complaint
- P23 Adolescent behav. Symptom/complt.
- P24 Specific learning problem
- P25 Phase of life problem adult
- P27 Fear of mental disorder
- P28 Limited function/disability (p)
- P29 Psychological symptom/complt other
- P70 Dementia
- P71 Organic psychosis other
- P72 Schizophrenia
- P73 Affective psychosis
- P74 Anxiety disorder/anxiety state
- P75 Somatization disorder
- P76 Depressive disorder
- P77 Suicide/suicide attempt
- P78 Neuraesthenia/surmenage
- P79 Phobia/compulsive disorder
- P80 Personality disorder
- P81 Hyperkinetic disorder
- P82 Post-traumatic stress disorder
- P85 Mental retardation
- P86 Anorexia nervosa/bulimia
- P98 Psychosis NOS/other
- P99 Psychological disorders, other

Respiratory R

- R01 Pain respiratory system
- R02 Shortness of breath/dyspnoea
- R03 Wheezing
- R04 Breathing problem, other
- R05 Cough
- R06 Nose bleed/epistaxis
- R07 Sneezing/nasal congestion
- R08 Nose symptom/complaint other
- R09 Sinus symptom/complaint
- R21 Throat symptom/complaint
- R23 Voice symptom/complaint
- R24 Haemoptysis
- R25 Sputum/phlegm abnormal
- R26 Fear of cancer respiratory system
- R27 Fear of respiratory disease, other
- R28 Limited function/disability (r)
- R29 Respiratory symptom/complaint oth.
- R71 Whooping cough
- R72 Strep throat
- R73 Boil/abscess nose
- R74 Upper respiratory infection acute
- R75 Sinusitis acute/chronic
- R76 Tonsillitis acute
- R77 Laryngitis/tracheitis acute
- R78 Acute bronchitis/bronchiolitis
- R79 Chronic bronchitis
- R80 Influenza
- R81 Pneumonia
- R82 Pleurisy/pleural effusion
- R83 Respiratory infection other
- R84 Malignant neoplasm bronchus/lung
- R85 Malinant neoplasm respiratory, other
- R86 Benign neoplasm respiratory
- R87 Foreign body nose/larynx/bronch
- R88 Injury respiratory other
- R89 Congenital anomaly respiratory
- R90 Hypertrophy tonsils/adenoids
- R92 Neoplasm respiratory unspecified
- R95 Chronic obstructive pulmonary dis
- R96 Asthma
- R97 Allergic rhinitis
- R98 Hyperventilation syndrome
- R99 Respiratory disease other

PROCESS CODES

SYMPTOMS/COMPLAINTS

INFECTIONS

NEOPLASMS

INJURIES

CONGENITAL ANOMALIES

OTHER DIAGNOSES

Skin S

- S01 Pain/tenderness of skin
- S02 Pruritus
- S03 Warts
- S04 Lump/swelling localized
- S05 Lumps/swellings generalized
- S06 Rash localized
- S07 Rash generalized
- S08 Skin colour change
- S09 Infected finger/toe
- S10 Boil/carbuncle
- S11 Skin infection post-traumatic
- S12 Insect bite/sting
- S13 Animal/human bite
- S14 Burn/scald
- S15 Foreign body in skin
- S16 Bruise/contusion
- S17 Abrasion/scratch/blister
- S18 Laceration/cut
- S19 Skin injury other
- S20 Corn/callosity
- S21 Skin texture symptom/complaint
- S22 Nail symptom/complaint
- S23 Hair loss/baldness
- S24 Hair/scalp symptom/complaint
- S26 Fear of cancer of skin
- S27 Fear of skin disease other
- S28 Limited function/disability (s)
- S29 Skin symptom/complaint other
- S70 Herpes zoster
- S71 Herpes simplex
- S72 Scabies/other acariasis
- S73 Pediculosis/skin infestation other
- S74 Dermatophytosis
- S75 Moniliasis/candidiasis skin
- S76 Skin infection other
- S77 Malignant neoplasm of skin
- S78 Lipoma
- S79 Neoplasm skin benign/unspecified
- S80 Solar keratosis/sunburn
- S81 Haemangioma/lymphangioma
- S82 Naevus/mole
- S83 Congenital skin anomaly other
- S84 Impetigo
- S85 Pilonidal cyst/fistula
- S86 Dermatitis seborrhoeic
- S87 Dermatitis/atopic eczema
- S88 Dermatitis contact/allergic
- S89 Diaper rash
- S90 Pityriasis rosea
- S91 Psoriasis
- S92 Sweat gland disease
- S93 Sebaceous cyst
- S94 Ingrowing nail
- S95 Molluscum contagiosum
- S96 Acne
- S97 Chronic ulcer skin
- S98 Urticaria
- S99 Skin disease, other

Endocrine/Metabolic and Nutritional T

- T01 Excessive thirst
- T02 Excessive appetite
- T03 Loss of appetite
- T04 Feeding problem of infant/child
- T05 Feeding problem of adult
- T07 Weight gain
- T08 Weight loss
- T10 Growth delay
- T11 Dehydration
- T26 Fear of cancer of endocrine system
- T27 Fear endocrine/metabolic dis other
- T28 Limited function/disability (t)
- T29 Endocrine/met./sympt/complt other
- T70 Endocrine infection
- T71 Malignant neoplasm thyroid
- T72 Benign neoplasm thyroid
- T73 Neoplasm endocrine oth/unspecified
- T78 Thyroglossal duct/cyst
- T80 Congenital anom endocrine/metab.
- T81 Goitre
- T82 Obesity
- T83 Overweight
- T85 Hyperthyroidism/thyrotoxicosis
- T86 Hypothyroidism/myxoedema
- T87 Hypoglycaemia
- T89 Diabetes insulin dependent
- T90 Diabetes non-insulin dependent
- T91 Vitamin/nutritional deficiency
- T92 Gout
- T93 Lipid disorder
- T99 Endocrine/metab/nutrit. dis. other

Urological U

- U01 Dysuria/painful urination
- U02 Urinary frequency/urgency
- U04 Incontinence urine
- U05 Urination problems other
- U06 Haematuria
- U07 Urine symptom/complaint other
- U08 Urinary retention
- U13 Bladder symptom/complaint other
- U14 Kidney symptom/complaint
- U26 Fear of cancer of urinary system
- U27 Fear of urinary disease other
- U28 Limited function/disability urinary
- U29 Urinary symptom/complaint other
- U70 Pyelonephritis/pyelitis
- U71 Cystitis/urinary infection other
- U72 Urethritis
- U75 Malignant neoplasm of kidney
- U76 Malignant neoplasm of bladder
- U77 Malignant neoplasm urinary other
- U78 Benign neoplasm urinary tract
- U79 Neoplasm urinary tract NOS
- U80 Injury urinary tract
- U85 Congenital anomaly urinary tract
- U88 Glomerulonephritis/nephrosis
- U90 Orthostatic albumin./proteinuria
- U95 Urinary calculus
- U98 Abnormal urine test NOS
- U99 Urinary disease, other

Pregnancy, Childbearing, Family Planning W

- W01 Question of pregnancy
- W02 Fear of pregnancy
- W03 Antepartum bleeding
- W05 Pregnancy vomiting/nausea
- W10 Contraception postcoital
- W11 Contraception oral
- W12 Contraception intrauterine
- W13 Sterilization
- W14 Contraception other
- W15 Infertility/subfertility
- W17 Post-partum bleeding
- W18 Post-partum symptom/complaint oth.
- W19 Breast/lactation symptom/complaint
- W21 Concern body image in pregnancy
- W27 Fear complications of pregnancy
- W28 Limited function/disability (w)
- W29 Pregnancy symptom/complaint other
- W70 Puerperal infection/sepsis
- W71 Infection complicating pregnancy
- W72 Malignant neoplasm relate to preg.
- W73 Benign/unspec. neoplasm/pregnancy
- W75 Injury complicating pregnancy
- W76 Congenital anomaly complicate preg.
- W78 Pregnancy
- W79 Unwanted pregnancy
- W80 Ectopic pregnancy
- W81 Toxaemia of pregnancy
- W82 Abortion spontaneous
- W83 Abortion induced
- W84 Pregnancy high risk
- W85 Gestational diabetes
- W90 Uncomplicate labour/delivery live
- W91 Uncomplicate labour/delivery still
- W92 Complicate labour/ delivery livebirth
- W93 Complicate labour/delivery stillbirth
- W94 Puerperal mastitis
- W95 Breast disorder in pregnancy other
- W96 Complications of puerperium other
- W99 Disorder pregnancy/delivery, other

Female Genital X

- X01 Genital pain female
- X02 Menstrual pain
- X03 Intermenstrual pain
- X04 Painful intercourse female
- X05 Menstruation absent/scanty
- X06 Menstruation excessive
- X07 Menstruation irregular/frequent
- X08 Intermenstrual bleeding
- X09 Premenstrual symptom/complaint
- X10 Postponement of menstruation
- X11 Menopausal symptom/complaint
- X12 Postmenopausal bleeding
- X13 Postcoital bleeding
- X14 Vaginal discharge
- X15 Vaginal symptom/complaint other
- X16 Vulval symptom/complaint
- X17 Pelvis symptom/complaint female
- X18 Breast pain female
- X19 Breast lump/mass female
- X20 Nipple symptom/complaint female
- X21 Breast symptom/complt. female other
- X22 Concern breast appearance female
- X23 Fear sexually transmitted disease (f)
- X24 Fear of sexual dysfunction female
- X25 Fear of genital cancer female
- X26 Fear of breast cancer female
- X27 Fear genital/breast disease other (f)
- X28 Limited function/disability (x)
- X29 Genital symptom/complt female oth.
- X70 Syphilis female
- X71 Gonorrhoea female
- X72 Genital candidiasis female
- X73 Genital trichomoniasis female
- X74 Pelvic inflammatory disease
- X75 Malignant neoplasm cervix
- X76 Malignant neoplasm breast female
- X77 Malignant neoplasm genital other (f)
- X78 Fibromyoma uterus
- X79 Benign neoplasm breast female
- X80 Benign neoplasm female genital
- X81 Genital neoplasm oth/unspecied (f)
- X82 Injury genital female
- X83 Congenital anomaly genital female
- X84 Vaginitis/vulvitis NOS
- X85 Cervical disease NOS
- X86 Abnormal cervix smear
- X87 Uterovaginal prolapse
- X88 Fibrocystic disease breast
- X89 Premenstrual tension syndrome
- X90 Genital herpes female
- X91 Condylomata acuminata female
- X92 Chlamydia infection genital (f)
- X99 Genital disease female, other

Male Genital Y

- Y01 Pain in penis
- Y02 Pain in testis/scrotum
- Y03 Urethral discharge
- Y04 Penis symptom/complaint other
- Y05 Scrotum/testis sympt/complt. other
- Y06 Prostate symptom/complaint
- Y07 Impotence NOS
- Y08 Sexual function sympt./complt.(m)
- Y10 Infertility/subfertility male
- Y13 Sterilization male
- Y14 Family planning male other
- Y16 Breast symptom/complaint male
- Y24 Fear of sexual dysfunction male
- Y25 Fear sexually transmitted dis. male
- Y26 Fear of qenital cancer male
- Y27 Fear of genital disease male other
- Y28 Limited function/disability (y)
- Y29 Genital sympt./complt.male other
- Y70 Syphilis male
- Y71 Gonorrhoea male
- Y72 Genital herpes male
- Y73 Prostatitis/seminal vesiculitis
- Y74 Orchitis/epididymitis
- Y75 Balanitis
- Y76 Condylomata acuminata male
- Y77 Malignant neoplasm prostate
- Y78 Malign neoplasm male genital other
- Y79 Benign/unspec. neoplasm gen. (m)
- Y80 Injury male genital
- Y81 Phimosis/redundant prepuce
- Y82 Hypospadias
- Y83 Undescended testicle
- Y84 Congenital genl anomaly (m) other
- Y85 Benign prostatic hypertrophy
- Y86 Hydrocoele
- Y99 Genital disease male, other

Social Problems Z

- Z01 Poverty/financial problem
- Z02 Food/water problem
- Z03 Housing/neighbourhood problem
- Z04 Social cultural problem
- Z05 Work problem
- Z06 Unemployment problem
- Z07 Education problem
- Z08 Social welfare problem
- Z09 Legal problem
- Z10 Health care system problem
- Z11 Compliance/being ill problem
- Z12 Relationship problem with partner
- Z13 Partner's behaviour problem
- Z14 Partner illness problem
- Z15 Loss/death of partner problem
- Z16 Relationship problem with child
- Z18 Illness problem with child
- Z19 Loss/death of child problem
- Z20 Relationship prob. parent/family
- Z21 Behaviour problem parent/family
- Z22 Illness problem parent/family
- Z23 Loss/death parent/family member
- Z24 Relationship problem friend
- Z25 Assault/harmful event problem
- Z27 Fear of a social problem
- Z28 Limited function/disability (z)
- Z29 Social problem NOS

Abbreviations

Anom	anomaly
behav.	behaviour
bronch.	bronchus
complicat.	complication
congen.	congenital
dis.	disease
eval.	evaluation
exam.	examination
gen.	genital
malig.	malignant
metab.	metabolic
musculo.	musculoskeletal
NEC	not elsewhere classified
NOS	not otherwise specified
nutrit.	nutrition
oth	other
preg.	pregnancy
prob.	problem
RFE	reason for encounter
sympt.	symptom
unspec.	unspecified
w	with
w/o	without

Supplementary Methods 2. ICPC-2 codes and prevalence estimates for mental-health conditions in the Norwegian study population (N=2,753,646).

Mental-health condition	ICPC-2 code/s	N (%)
Acute stress reaction	P02 Acute stress reaction	561,420 (20.4)
ADHD	P81 Hyperkinetic disorder	64,192 (2.3)
Anxiety	P01 Feeling anxious/nervous/tense<br>P74 Anxiety disorder/anxiety state	376,854 (13.7)
Chronic fatigue	P78 Neuraesthenia/surmenage	35,095 (1.3)
Depression	P03 Feeling depressed<br>P76 Depressive disorder	700,261 (25.4)
Developmental delay/learning problem	P24 Specific learning problem<br>P28 Limited function/disability (p)<br>P85 Mental retardation	38,701 (1.4)
Personality disorder	P80 Personality disorder	41,472 (1.5)
Phobia/compulsive disorder	P79 Phobia/compulsive disorder	85,309 (3.1)
Psychosis	P71 Organic psychosis other<br>P72 Schizophrenia<br>P73 Affective psychosis<br>P98 Psychosis NOS/other	80,248 (2.9)
PTSD	P82 Post-traumatic stress disorder	48,030 (1.7)
Sexual concerns	P07 Sexual desire reduced<br>P08 Sexual fulfilment reduced<br>P09 Sexual preference concern	72,297 (2.6)
Sleep disturbance	P06 Sleep disturbance	495,562 (18.0)
Substance abuse	P15 Chronic alcohol abuse<br>P16 Acute alcohol abuse<br>P17 Tobacco abuse<br>P18 Medication abuse<br>P19 Drug abuse	192,488 (7.0)
Psychological condition, not otherwise specified (NOS)	P29 Psychological symptom/complaint other<br>P99 Psychological disorders, other	497,001 (18.0)

Note. Prevalences were calculated across the 14-year observation period from January 2006–December 2019.

Supplementary Methods 3. ICPC-2 codes and prevalence estimates for injuries in the Norwegian study population (N=2,753,646).

Injury	ICPC-2 code/s	N (%)
A. General and Unspecified	A80 Trauma/injury NOS<br>A81 Multiple trauma/injuries<br>A82 Secondary effect of trauma<br>A84 Poisoning by a medical agent<br>A85 Adverse effect medical agent<br>A86 Toxic effect non-medicinal substance<br>A87 Complication of medical treatment<br>A88 Adverse effect physical factor<br>A89 Effect prosthetic device	544,656 (19.8)
D. Digestive	D79 Foreign body digestive system<br>D80 Injury digestive system other	32,718 (1.2)
F. Eye	F75 Contusion/haemorrhave eye<br>F76 Foreign body in eye<br>F79 Injury eye other	184,560 (6.7)
H. Ear	H76 Foreign body in ear<br>H77 Perforation ear drum<br>H78 Superficial injury of ear<br>H79 Ear injury other	36,699 (1.3)
L. Musculoskeletal	L72 Fracture: radius/ulna<br>L73 Fracture: tibia/fibula<br>L74 Fracture: hand/foot bone<br>L75 Fracture: femur<br>L76 Fracture: other<br>L77 Sprain/strain of ankle<br>L78 Sprain/strain of knee<br>L79 Sprain/strain of join NOS<br>L80 Dislocation/subluxation<br>L81 Injury musculoskeletal NOS<br>L96 Acute internal damage knee	1,066,371 (38.7)
N. Neurological	N79 Concussion<br>N80 Head injury other	176,984 (6.4)
S. Skin	S12 Insect bite/sting<br>S13 Animal/human bite<br>S14 Burn/scald<br>S15 Foreign body in skin<br>S16 Bruise/contusion<br>S17 Abrasion/scratch/blister<br>S18 Laceration/cut<br>S19 Skin injury other	875,558 (31.8)
Other[a]<br>B. Blood, Blood Forming Organs and Immune Mechanism<br><br>R. Respiratory<br><br>U. Urological<br><br>W. Pregnancy, Childbearing, Family Planning	 B76 Ruptured spleen traumatic<br>B77 Injury blood/lymph/spleen other<br><br>R87 Foreign body nose/larynx/bronch<br>R88 Injury respiratory other<br><br>U80 Injury urinary tract<br><br>W75 Injury complicating pregnancy	30,413 (1.1)

X/Y. Female/Male Genital	X82 Injury genital female<br>Y80 Injury male genital	

Note. Because injuries are distributed throughout different chapters in the ICPC-2 according to the body system they involve, we pooled the relevant codes across the different chapters. Prevalences were calculated across the 14-year observation period from January 2006–December 2019.

[a]Other injuries (including injuries in chapters B, R, U, W, and X/Y) were excluded from analyses as they were highly heterogeneous and accounted for only 1.1% of all injuries.

Supplementary Methods 4. Ascertainment of mental-health conditions in the NZIDI study population (N=2,238,813), using publicly funded inpatient-hospital records.

Our ascertainment scheme for mental-health conditions is previously published.[1,2] Briefly, mental-health conditions were ascertained using records of diagnoses made in public hospitals, based on the International Classification of Diseases, 9th Revision (ICD-9, for admissions between July 1989–June 1999) and 10th Revision (ICD-10, for admissions between July 1999–June 2019). We obtained information about nine broad categories of mental-health conditions using the primary diagnoses for each hospital admission, based on ICD-10 and corresponding ICD-9 codes. The ICD-10 code ranges for each diagnostic category and their prevalences in the current study population during the 30-year observation period from June 1989-July 2019 are as follows:

Mental and behavioral disorders due to psychoactive substance use ("substance use disorders"): F10-F19, 1.0%;
Schizophrenia and related disorders ("psychotic disorders"): F20-F29, 0.9%;
Mood disorders: F30-F39, 1.3%;
Neurotic, stress-related, and somatoform disorders ("neurotic disorders"): F40-F48, 0.9%;
Syndromes associated with physiological disturbances and physical factors ("physiological-disturbance disorders"): F50-F59, 0.1%;
Disorders of adult personality and behavior ("personality disorders"): F60-F69, 0.2%;
Pervasive and specific developmental disorders ("developmental disorders"): F80-F89, 0.01%;
Behavioral and emotional disorders with onset usually occurring in childhood and adolescence ("behavioral disorders"): F90-F98, 0.03%;
Unspecified mental disorders: F99, 0.1%

To achieve consistency across diagnostic schemes, corresponding diagnoses in the ICD-9 were ascertained using mapped codes provided by the New Zealand Ministry of Health.[1,2] Mapping code is hosted at the following website: https://moffittcaspi.trinity.duke.edu/research-topics/statistical-code.

Supplementary Methods 5. Ascertainment of injuries in the NZIDI study population (N=2,238,813), using publicly funded inpatient-hospital records.

We used records of diagnoses made in public hospitals, based on the International Classification of Diseases, 9th and 10th Revisions (ICD-9 and ICD-10). We ascertained all hospital events with a primary injury diagnosis, excluding complications of surgical and medical care, not elsewhere classified (code ranges included in analyses: ICD-9=800-995, ICD-10=S00-T79). We classified injuries according to body region using the Barell Matrix[3] and according to injury intent using the external cause-of-injury matrix developed by the National Center for Injury Prevention and Control.[3] We classified intent into: self-harm, assault, and unintentional; other intent (i.e., legal intervention or war); and events of undetermined intent. To achieve consistency in injury classification across diagnostic schemes, ICD-10 codes were mapped to their corresponding ICD-9 codes, using mapped codes provided by the New Zealand Ministry of Health. Mapping code is hosted at the following website: https://moffittcaspi.trinity.duke.edu/research-topics/statistical-code.

Prevalences of the different injury intents in the current study population, across the 30-year observation period from June 1989-July 2019, were as follows:

Unintentional injury = 19.2%
Assault injury = 1.6%
Intentional self-harm = 1.5%
Other intent = 0.02%
Undetermined intent = 0.4%

Supplementary Methods 6. Ascertainment of injuries in the NZIDI study population (N=2,159,814[a]), using Accident Compensation Corporation records.

We used information about insurance claims for injuries, including treatment and entitlement claims, from records maintained by the Accident Compensation Corporation (ACC), the national provider of comprehensive, no-fault personal injury coverage for New Zealanders. All New Zealanders who have experienced an injury are eligible for coverage, regardless of how the injury occurred and regardless of the setting in which it was treated. ACC claims capture less-severe injuries, more severe hospital-treated cases, and long-term injuries for which hospital treatment is not appropriate (e.g., severe sprains). ACC covers much of the costs of emergency provision in hospitals, all or some of the costs of immediate care and rehabilitation, and costs to the person associated with income maintenance or life adaptations.[4] Whereas inpatient-hospital records were available for the full 30-year observation period (July 1989 to June 2019), ACC records were available for the 19-year period from July 2000 to June 2019.

[a]Of the 2,238,813 individuals in the NZIDI study population, 2,159,814 (96.5%) were still alive in July 2000 when Accident Compensation Corporation records became available and resided in the country for any time during the 19-year ACC observation period (July 2000-June 2019).

Supplementary Methods 7. Random-matching procedure in the NZIDI study population.

Our random-matching procedure is previously published.[1,2] In the NZIDI, we assessed whether hospital admissions with a primary mental-health diagnosis were associated with subsequent hospital admissions with a primary injury diagnosis during the July 1989-June 2019 observation period. Our analysis needed to account for the different duration of observation time among cases (those with a mental-health condition, who were observed from their first mental-health admission, which could occur at any time during the study period) and controls (those without a mental-health admission, whose observation time was the full 30 years). Failing to account for differing observation periods could lead to biased estimates, because controls would have more time to receive an injury diagnosis than cases.

To address this problem, we randomly assigned observation periods to controls to match the observation periods of cases, based on the distributions of admission dates for cases' mental-health admissions. For example, suppose that .53%, .37%, and .41% of cases had their first mental-health admission in July 1992, March 1998, and October 2015, respectively. In this scenario, we would randomly assign .53% of controls to have their observation period start in July 1992, .37% to have their observation period start in March 1998, and .41% to have their observation period start in October 2015. Importantly, we only recorded hospital admissions with a primary injury diagnosis during these truncated observation periods, ignoring all diagnoses prior to the randomly-assigned starting months. We took this approach for each of the 360 months from July 1989 to June 2019, and so created a cohort of controls with the same distribution of observation periods as cases.

To account for potentially different distributions of mental-health admissions across age and sex, we used birth cohort- and sex-specific distributions of start dates among cases to randomly assign start dates to controls. That is, the distribution of start dates among male cases born in 1929-39 was used to randomly assign start dates to male controls born in 1929-39, the distribution of start dates among female cases born in 1940-49 was used to randomly assign start dates to female controls born in 1940-49, etc.

For analyses of ACC injury-claims data, for which the observation period started in July 2000, we randomly matched controls to cases based on cases' first mental-health admission during the July 2000–June 2019 observation period.

Supplementary Table 1. Risk differences [and 95% confidence intervals] for monthly risk of injuries as a function of presenting to primary care for a mental-health condition, in the Norwegian study population. Estimates indicate percentage-point differences. Inverse probability weights were used to balance mental-health groups on age at baseline, sex, county of residence, and educational attainment. Confidence intervals correspond to estimates reported in Figure 1, Panel B in the main text.

Any mental health condition	0.67 [0.67, 0.67]
Acute stress reaction	0.77 [0.77, 0.78]
ADHD	1.25 [1.22, 1.27]
Anxiety	0.88 [0.87, 0.88]
Chronic fatigue	0.75 [0.74, 0.77]
Depression	0.82 [0.82, 0.83]
Developmental delay/learning problem	0.85 [0.83, 0.88]
Personality disorder	1.28 [1.26, 1.30]
Phobia/compulsive disorder	0.69 [0.68, 0.70]
Psychosis	0.97 [0.96, 0.98]
PTSD	1.84 [1.81, 1.86]
Sexual concern	0.73 [0.70, 0.76]
Sleep disturbance	1.00 [1.00, 1.01]
Substance abuse	1.37 [1.37, 1.38]
Psychological condition NOS	0.76 [0.76, 0.77]

Supplementary Table 2. Adjusted hazard ratios [and 95% confidence intervals] for associations of mental-health conditions with subsequent injuries in Norwegian primary-care records, before and after accounting for education.

	Adjusted for age at baseline, sex, county of residence, and prior injury	Further adjusted for educational attainment
Total study population	2.07 [2.06, 2.08]	1.80 [1.80, 1.81]
Men		
Born 1986-96	2.25 [2.22, 2.28]	1.95 [1.93, 1.97]
Born 1976-86	2.19 [2.17, 2.22]	1.93 [1.91, 1.95]
Born 1966-76	2.12 [2.10, 2.15]	1.84 [1.82, 1.86]
Born 1956-66	2.14 [2.11, 2.17]	1.87 [1.85, 1.89]
Born 1946-56	2.11 [2.08, 2.14]	1.85 [1.83, 1.87]
Women		
Born 1986-96	2.57 [2.53, 2.60]	2.21 [2.19, 2.24]
Born 1976-86	2.13 [2.09, 2.16]	1.83 [1.81, 1.86]
Born 1966-76	1.92 [1.90, 1.95]	1.66 [1.65, 1.68]
Born 1956-66	1.86 [1.84, 1.88]	1.64 [1.63, 1.66]
Born 1946-56	1.87 [1.84, 1.89]	1.66 [1.64, 1.67]

Note. Inverse probability weights were used to account for effects due to age at baseline (in January 2006), sex, county of residence, and educational attainment. For educational attainment, we used the Norwegian Standard Classification of Education with nine levels (coded 0-8). For individuals over age 30, we used their own education. Younger individuals were assigned the maximum of either their mother’s or father’s educational attainment.

Supplementary Table 3. Adjusted risk ratios [and 95% confidence intervals] for associations of mental-health conditions with subsequent self-harm, assault, and unintentional injuries in New Zealand inpatient-hospital records, before and after accounting for neighborhood socioeconomic deprivation.

	Adjusted for birth year, sex, and prior injury			Further adjusted for neighborhood deprivation		
	Self-harm injury	Assault injury	Unintentional injury	Self-harm injury	Assault injury	Unintentional injury
Total study population	25.14 [24.38, 25.91]	5.68 [5.47, 5.90]	2.17 [2.13, 2.20]	21.51 [20.86, 22.17]	4.57 [4.40, 4.75]	1.91 [1.88, 1.94]
Men						
Born 1970-79	19.53 [18.16, 21.01]	4.25 [3.97, 4.55]	1.79 [1.72, 1.86]	17.36 [16.13, 18.69]	3.55 [3.32, 3.80]	1.65 [1.59, 1.71]
Born 1960-69	27.62 [25.49, 29.93]	5.36 [4.97, 5.79]	1.87 [1.80, 1.95]	23.88 [22.01, 25.90]	4.30 [3.98, 4.64]	1.72 [1.66, 1.79]
Born 1950-59	34.41 [30.82, 38.41]	7.05 [6.25, 7.95]	2.00 [1.91, 2.10]	29.30 [26.21, 32.75]	5.41 [4.80, 6.10]	1.82 [1.73, 1.90]
Born 1940-49	39.36 [32.60, 47.52]	7.91 [6.29, 9.96]	2.09 [1.97, 2.10]	33.45 [27.65, 40.46]	5.97 [4.74, 7.51]	1.88 [1.77, 2.00]
Born 1929-39	23.49 [17.54, 31.46]	5.62 [3.47, 9.09]	1.77 [1.65, 1.91]	20.53 [15.33, 27.51]	4.40 [2.72, 7.11]	1.55 [1.44, 1.67]
Women						
Born 1970-79	16.87 [15.81, 17.99]	6.22 [5.62, 6.88]	2.64 [2.52, 2.77]	15.55 [14.58, 16.59]	5.31 [4.80, 5.86]	2.47 [2.36, 2.59]
Born 1960-69	26.80 [25.03, 28.71]	7.55 [6.69, 8.53]	2.71 [2.52, 2.77]	22.66 [21.16, 24.27]	5.73 [5.09, 6.46]	2.36 [2.26, 2.47]
Born 1950-59	35.61 [32.27, 39.29]	11.32 [9.34, 13.73]	2.93 [2.77, 3.10]	25.81 [23.40, 28.47]	7.43 [6.15, 8.97]	2.28 [2.16, 2.41]
Born 1940-49	47.71 [40.45, 56.27]	10.24 [7.01, 14.96]	2.74 [2.58, 2.91]	34.86 [29.52, 41.14]	6.42 [4.42, 9.33]	2.04 [1.92, 2.16]
Born 1929-39	37.32 [28.63, 48.66]	3.59 [1.55, 8.35]	1.83 [1.72, 1.95]	28.37 [21.74, 37.02]	2.62 [1.13, 6.03]	1.43 [1.34, 1.52]

Note. Neighborhood socioeconomic deprivation was assessed using the 2013 New Zealand Deprivation Index (NZDep2013), an area-based measure of socioeconomic disadvantage derived from nine Census variables capturing area-level rates of unemployment, education, homeownership, and other domains.[5] The NZDep2013 assigns Census areas ("small areas" with a population of at least 100 people) a deprivation decile value ranging from 1 (least deprived) to 10 (most deprived). Following prior work,[6] decile scores were converted to quintiles for analysis. Neighborhood-deprivation information was available from 2000 to the end of the study period (2019). We used deprivation information for individuals' first registered address during the period. Of the 2,238,813 individuals in the NZIDI study population, 338,667 (15.1%) were missing neighborhood-deprivation information. They were included in analyses by coding missingness as a variable category. All models controlled for birth year and for injuries diagnosed before individuals' index mental-health condition. Models estimated within the total study population also controlled for sex. Mental-health cases and controls were matched on observation time.

Supplementary Table 4. Adjusted risk ratios [and 95% confidence intervals] for associations of mental-health conditions with subsequent injuries in the NZIDI study population, by type of mental-health condition.

	Substance-use disorder	Psychotic disorder	Mood disorder	Neurotic (anxiety) disorder
Total study population	2.68 [2.61, 2.75]	2.10 [2.03, 2.17]	2.23 [2.18, 2.29]	2.17 [2.11, 2.24]
Men				
Born 1970-79	2.27 [2.16, 2.40]	1.69 [1.58, 1.82]	1.80 [1.68, 1.93]	1.85 [1.71, 2.01]
Born 1960-69	2.49 [2.35, 2.63]	1.82 [1.70, 1.96]	1.87 [1.74, 2.00]	1.80 [1.65, 1.96]
Born 1950-59	2.57 [2.39, 2.77]	2.01 [1.83, 2.20]	1.91 [1.76, 2.08]	1.96 [1.78, 2.16]
Born 1940-49	2.75 [2.50, 3.03]	2.08 [1.80, 2.40]	1.89 [1.70, 2.10]	1.85 [1.62, 2.11]
Born 1929-39	2.21 [1.95, 2.50]	1.71 [1.39, 2.10]	1.71 [1.50, 1.94]	1.50 [1.28, 1.76]
Women				
Born 1970-79	3.37 [3.13, 3.62]	2.45 [2.19, 2.75]	2.78 [2.58, 2.99]	2.87 [2.64, 3.13]
Born 1960-69	3.54 [3.26, 3.84]	2.70 [2.45, 2.98]	2.87 [2.67, 3.08]	2.88 [2.65, 3.13]
Born 1950-59	3.57 [3.19, 4.01]	3.08 [2.77, 3.42]	3.12 [2.87, 3.39]	2.69 [2.42, 2.98]
Born 1940-49	3.25 [2.81, 3.75]	2.70 [2.37, 3.08]	2.96 [2.71, 3.25]	2.38 [2.12, 2.66]
Born 1929-39	2.10 [1.76, 2.50]	1.74 [1.49, 2.03]	1.86 [1.69, 2.05]	1.72 [1.55, 1.90]

Note. Models controlled for birth year and for injuries diagnosed before the index mental-health condition. Models estimated within the total study population also controlled for sex. Mental-health cases and controls were matched on observation time. Self-harm injuries were excluded from the outcome.

Supplementary Table 5. Adjusted risk ratios [and 95% confidence intervals] for associations of mental-health conditions with subsequent injuries to different body regions, in the NZIDI study population.

	Traumatic brain injury	Other head, face, and neck	Torso	Spine and back	Extremities	Unclassified
Total study population	4.00 [3.87, 4.15]	3.71 [3.59, 3.84]	2.96 [2.86, 3.07]	2.23 [2.11, 2.36]	1.99 [1.95, 2.03]	8.06 [7.84, 8.28]
Men						
Born 1970-79	3.54 [3.28, 3.81]	2.84 [2.64, 3.05]	2.65 [2.42, 2.89]	1.89 [1.65, 2.16]	1.69 [1.62, 1.77]	6.84 [6.41, 7.30]
Born 1960-69	3.97 [3.67, 4.30]	3.60 [3.34, 3.88]	2.73 [2.51, 2.97]	1.88 [1.64, 2.16]	1.72 [1.64, 1.80]	6.81 [6.37, 7.28]
Born 1950-59	4.42 [4.00, 4.89]	3.83 [3.46, 4.24]	2.74 [2.47, 3.04]	2.07 [1.75, 2.46]	1.73 [1.63, 1.84]	7.06 [6.50, 7.66]
Born 1940-49	3.84 [3.34, 4.40]	4.07 [3.56, 4.66]	2.75 [2.41, 3.14]	2.31 [1.86, 2.87]	1.91 [1.76, 2.06]	5.15 [4.55, 5.83]
Born 1929-39	2.51 [2.11, 2.97]	2.66 [2.27, 3.11]	2.08 [1.77, 2.45]	1.41 [1.01, 1.97]	1.73 [1.56, 1.90]	3.26 [2.73, 3.90]
Women						
Born 1970-79	4.47 [4.03, 4.96]	4.78 [4.31, 5.31]	3.34 [2.99, 3.74]	2.70 [2.31, 3.17]	2.44 [2.30, 2.59]	10.30 [9.58, 11.07]
Born 1960-69	4.93 [4.42, 5.51]	4.70 [4.19, 5.27]	4.03 [3.61, 4.50]	2.94 [2.50, 3.44]	2.49 [2.35, 2.64]	11.97 [11.12, 12.88]
Born 1950-59	5.08 [4.40, 5.86]	6.06 [5.25, 6.99]	4.20 [3.66, 4.81]	2.85 [2.30, 3.52]	2.62 [2.44, 2.81]	13.27 [12.07, 14.59]
Born 1940-49	4.70 [4.00, 5.53]	4.80 [4.10, 5.62]	3.93 [3.39, 4.57]	2.63 [2.03, 3.41]	2.50 [2.31, 2.69]	10.05 [8.92, 11.32]
Born 1929-39	2.27 [1.90, 2.70]	2.57 [2.24, 2.96]	1.92 [1.65, 2.23]	1.87 [1.46, 2.38]	1.80 [1.67, 1.94]	4.04 [3.43, 4.76]

Note. Models controlled for birth year and for injuries diagnosed before the index mental-health condition. Models estimated within the total study population also controlled for sex. Mental-health cases and controls were matched on observation time. Self-harm injuries were excluded from the outcome.

Supplementary Table 6. Adjusted risk ratios for associations of mental-health conditions with subsequent injuries across varying follow-up periods, in the NZIDI study population.

Follow-up interval	Risk ratio [95% confidence interval]
1 year	3.66 [3.51, 3.82]
1-5 years	2.79 [2.72, 2.86]
5-10 years	2.61 [2.54, 2.68]
10-15 years	2.55 [2.47, 2.63]
15-20 years	2.54 [2.45, 2.63]
20-25 years	2.54 [2.43, 2.65]
25-30 years	2.45 [2.30, 2.61]

Note. Models included all individuals with an injury admission in a given follow-up interval, including those who had an admission in a prior interval. Thus, individuals could contribute information about injury risk in more than one interval. Models controlled for birth year, sex, and injuries diagnosed before the index mental-health condition. Mental-health cases and controls were matched on observation time. Self-harm injuries were excluded from the outcome.

Supplementary Table 7. Adjusted risk ratios and incidence rate ratios for associations of mental-health conditions with subsequent injuries, with injuries ascertained from ACC claims data, in the NZIDI study population.

7A. Risk ratios [and 95% confidence intervals] for associations with any injury

	Baseline	Adjusted for neighborhood socioeconomic deprivation
Total study population	1.14 [1.13, 1.15]	1.08 [1.07, 1.09]
Men		
Born 1970-79	1.15 [1.12, 1.18]	1.11 [1.09, 1.14]
Born 1960-69	1.12 [1.09, 1.14]	1.09 [1.06, 1.11]
Born 1950-59	1.10 [1.07, 1.14]	1.06 [1.03, 1.10]
Born 1940-49	1.08 [1.04, 1.13]	1.03 [0.99, 1.08]
Born 1929-39	1.08 [1.02, 1.15]	1.00 [0.94, 1.06]
Women		
Born 1970-79	1.15 [1.12, 1.18]	1.12 [1.09, 1.15]
Born 1960-69	1.15 [1.12, 1.18]	1.10 [1.07, 1.13]
Born 1950-59	1.19 [1.15, 1.23]	1.08 [1.05, 1.12]
Born 1940-49	1.15 [1.10, 1.20]	1.03 [0.98, 1.07]
Born 1929-39	1.11 [1.06, 1.17]	1.01 [0.96, 1.06]

7B. Incidence rate ratios [and 95% confidence intervals] for associations with number of injuries

	Baseline	Adjusted for neighborhood socioeconomic deprivation
Total study population	1.35 [1.33, 1.36]	1.26 [1.24, 1.27]
Men		
Born 1970-79	1.19 [1.15, 1.22]	1.14 [1.11, 1.17]
Born 1960-69	1.20 [1.17, 1.24]	1.17 [1.14, 1.21]
Born 1950-59	1.19 [1.15, 1.23]	1.14 [1.10, 1.18]
Born 1940-49	1.18 [1.12, 1.24]	1.11 [1.06, 1.16]
Born 1929-39	1.06 [1.00, 1.13]	0.96 [0.90, 1.01]
Women		
Born 1970-79	1.51 [1.46, 1.55]	1.48 [1.44, 1.52]
Born 1960-69	1.48 [1.43, 1.53]	1.38 [1.34, 1.43]
Born 1950-59	1.69 [1.61, 1.77]	1.41 [1.35, 1.46]
Born 1940-49	1.61 [1.51, 1.71]	1.28 [1.21, 1.34]
Born 1929-39	1.30 [1.22, 1.38]	1.07 [1.01, 1.13]

Note. Models controlled for birth year and for ACC injury-insurance claims registered before the index mental-health condition. Models estimated within the total study population also controlled for sex. Mental-health cases and controls were matched on observation time.

Supplementary References

1. Richmond-Rakerd LS, D'Souza S, Milne BJ, Caspi A, Moffitt TE. Longitudinal associations of mental disorders with physical diseases and mortality among 2.3 million New Zealand citizens. *JAMA Netw Open*. 2021;4(1):e2033448.

2. Richmond-Rakerd LS, D'Souza S, Milne BJ, Caspi A, Moffitt TE. Longitudinal Associations of Mental Disorders With Dementia: 30-Year Analysis of 1.7 Million New Zealand Citizens. *JAMA Psychiatry*. 2022;79(4):333-340.

3. Injury Data and Resources - ICD Injury Matrices. October 12, 2021. Accessed June 25, 2022. https://www.cdc.gov/nchs/injury/injury_matrices.htm

4. Accident Compensation Corporation. Accident services: A guide for DHB and ACC staff. 2018. https://www.acc.co.nz/assets/provider/accident-services-a-guide-for-dhb-and-acc-staff.pdf

5. Atkinson J, Salmond C, Crampton P. NZDep2013 index of deprivation. University of Otago, Wellington. May 2014:1-64.

6. Reuben A, Richmond-Rakerd LS, Milne B, et al. Dementia, dementia's risk factors and premorbid brain structure are concentrated in disadvantaged areas: National register and birth-cohort geographic analyses. *Alzheimers Dement.* 2024;20:3167-3178.
