# Supplementary material for: Mental health conditions are associated with increased risk of subsequent self-harm, assault and unintentional injuries in two nations
Source: Nat Ment Health. 2025 Dec 22;4(1):102–11. doi: 10.1038/s44220-025-00553-w (PMC12789009; doi:10.1038/s44220-025-00553-w)
Supplement: Supplementary file 2 — Reporting Summary [file 44220_2025_553_MOESM2_ESM.pdf]

nature portfolio

Corresponding author(s): Leah Richmond-Rakerd
Last updated by author(s): Oct 22, 2025

Reporting Summary

Nature Portfolio wishes to improve the reproducibility of the work that we publish. This form provides structure for consistency and transparency in reporting. For further information on Nature Portfolio policies, see our Editorial Policies and the Editorial Policy Checklist.

Statistics

For all statistical analyses, confirm that the following items are present in the figure legend, table legend, main text, or Methods section.

n/a	Confirmed	
☐	☒	The exact sample size (n) for each experimental group/condition, given as a discrete number and unit of measurement
☐	☒	A statement on whether measurements were taken from distinct samples or whether the same sample was measured repeatedly
☐	☒	The statistical test(s) used AND whether they are one- or two-sided<br>*Only common tests should be described solely by name; describe more complex techniques in the Methods section.*
☐	☒	A description of all covariates tested
☒	☐	A description of any assumptions or corrections, such as tests of normality and adjustment for multiple comparisons
☐	☒	A full description of the statistical parameters including central tendency (e.g. means) or other basic estimates (e.g. regression coefficient) AND variation (e.g. standard deviation) or associated estimates of uncertainty (e.g. confidence intervals)
☐	☒	For null hypothesis testing, the test statistic (e.g. F, t, r) with confidence intervals, effect sizes, degrees of freedom and P value noted<br>*Give P values as exact values whenever suitable.*
☒	☐	For Bayesian analysis, information on the choice of priors and Markov chain Monte Carlo settings
☒	☐	For hierarchical and complex designs, identification of the appropriate level for tests and full reporting of outcomes
☐	☒	Estimates of effect sizes (e.g. Cohen's d, Pearson's r), indicating how they were calculated

Our web collection on statistics for biologists contains articles on many of the points above.

Software and code

Policy information about availability of computer code

Data collection	No software was used.
Data analysis	Analyses were performed in SAS version 9.4 and R version 4.2.3. Statistical code is available at https://github.com/leahrr-umich/MentalHealth-Injury.git.

For manuscripts utilizing custom algorithms or software that are central to the research but not yet described in published literature, software must be made available to editors and reviewers. We strongly encourage code deposition in a community repository (e.g. GitHub). See the Nature Portfolio guidelines for submitting code & software for further information.

Data

Policy information about availability of data

All manuscripts must include a data availability statement. This statement should provide the following information, where applicable:

- Accession codes, unique identifiers, or web links for publicly available datasets
- A description of any restrictions on data availability
- For clinical datasets or third party data, please ensure that the statement adheres to our policy

The data for this study are primary-care records for entire cohorts of the Norwegian population, and nationwide-register data from the New Zealand Integrated Data Infrastructure (NZIDI). The data cannot be shared by the authors because access to the data is regulated via managed-access programs. Researchers can access the Norwegian primary-care data by application to the Regional Committees for Medical and Health Research Ethics and the data owners (Statistics Norway and the

Norwegian Directorate of Health). Researchers who wish to use the NZIDI data must submit an application through Statistics New Zealand. Researchers can contact this report's authors if they have questions concerning the data.

Research involving human participants, their data, or biological material

Policy information about studies with human participants or human data. See also policy information about sex, gender (identity/presentation), and sexual orientation and race, ethnicity and racism.

Reporting on sex and gender	Norwegian study population: N = 2,753,646, 51.1% male. We report analyses for the full population and stratified by sex.<br><br>New Zealand Integrated Data Infrastructure (NZIDI) study population: N=2,238,813, 50.3% male. We report analyses for the full population and stratified by sex.
Reporting on race, ethnicity, or other socially relevant groupings	We report analyses in the total study populations, and stratified by age and sex.<br><br>In the Norwegian study population, mental-health groups were balanced on age at baseline, sex, county of residence, and education level. In the NZIDI study population, models controlled for birth year, sex, and neighborhood socioeconomic deprivation.<br><br>Information about ethnicity is not available in Norwegian administrative registers. We report ethnicity information for the NZIDI study population (see next box).
Population characteristics	Data about primary-care contacts for the entire nation of Norway were obtained for all individuals who were born in Norway between 1946-1996, resided in Norway throughout the January 2006-December 2019 observation period, and had information on variables used to construct inverse probability weights (N=2,753,646, 51.1% male, age at baseline=10-60y, age at end of 14-year observation=24-74y).<br><br>We obtained data for all individuals born in New Zealand between 1929-1979 who resided in New Zealand for any time during the July 1989-June 2019 observation period (N=2,238,813, 50.3% male, age at baseline=10-60y, age at end of 30-year observation=40-90y). Of the NZIDI study population, 1,692,612 individuals had ethnicity information, of whom 64.6% identified as European, 13.9% as Māori, 7.4% as Pacific, 10.5% as Asian, 2.4% as Middle Eastern, Latin American, and African (MELAA), and 1.3% as Other. Individuals could identify with more than one ethnic group. Ethnicity data are collated from multiple ranked data sources, with Census data given the highest priority; individuals are assigned the ethnic profile from the highest-ranked source available for them.
Recruitment	Nationwide databases of routinely-collected administrative data that are de-identified and made available for research.
Ethics oversight	Regional Committee for Research Ethics South East Norway (REK South East; 2018/434), Arts and Science Institutional Review Board at Duke University (2022-0260), University of Auckland Human Participants Ethics Committee (Ref. UAHPEC20738).

Note that full information on the approval of the study protocol must also be provided in the manuscript.

Field-specific reporting

Please select the one below that is the best fit for your research. If you are not sure, read the appropriate sections before making your selection.

☐ Life sciences ☒ Behavioural & social sciences ☐ Ecological, evolutionary & environmental sciences

For a reference copy of the document with all sections, see nature.com/documents/nr-reporting-summary-flat.pdf

Behavioural & social sciences study design

All studies must disclose on these points even when the disclosure is negative.

Study description	Quantitative analysis of data collected in population-based administrative registers.
Research sample	This representative population-based study included:<br><br>N = 2,753,646 individuals (51.1% male) who were born in Norway between 1946-1996, resided in Norway throughout the January 2006-December 2019 observation period, and had information on variables used to construct inverse probability weights (age at baseline=10-60y, age at end of 14-year observation=24-74y). Data were from a register of primary-care contacts for the entire nation of Norway.<br><br>N = 2,238,813 individuals (50.3% male) who were born in New Zealand between 1929-1979 and resided in New Zealand for any time during the July 1989-June 2019 observation period (age at baseline=10-60y, age at end of 30-year observation=40-90y). Data were from the New Zealand Integrated Data Infrastructure (IDI). More information about the IDI is located here: https://www.stats.govt.nz/integrated-data/integrated-data-infrastructure/.
Sampling strategy	Population-based administrative registers. No sample-size calculation was performed because the study populations comprised the Norwegian and New Zealand populations with the birth year and residency characteristics of interest, and (for Norway) with

	information on variables used to construct inverse probability weights. We did not subsample from these individuals. See "data exclusions" below.
Data collection	Norway: Analysis of medical records. All residents of Norway are assigned a primary-care physician (PCP). To be reimbursed for services, primary-care physicians bill the Norwegian Health Economics Administration and indicate at least one primary diagnosis or reason for a patient's visit.<br><br>New Zealand: Data were from the New Zealand Integrated Data Infrastructure (NZIDI), a collection of de-identified, individually-linked, whole-of-population administrative data sources.<br><br>The researchers were not involved in data collection. Researchers were not blinded to experimental conditions as this analysis comprised secondary data and participants were not allocated into experimental groups.
Timing	Norway: Observation period = January 2006-December 2019<br><br>New Zealand: Observation period = July 1989-June 2019
Data exclusions	Our Norwegian study population included all individuals who were born in Norway between 1946-1996, resided in Norway throughout the January 2006-December 2019 observation period, and had information on variables used to construct inverse probability weights. N = 845 individuals were excluded due to missing data on county of residence and/or education, leaving a final study population of N = 2,753,646 (51.1% male).<br><br>Our NZIDI study population included the 2,238,813 individuals (50.3% male) who were born in New Zealand between 1929-1979 and resided in New Zealand for any time during the July 1989-June 2019 observation period.
Non-participation	There was no non-participation. Our study comprised analyses of routinely-collected administrative data.
Randomization	There was no randomization. Participants were not allocated into experimental groups.

Reporting for specific materials, systems and methods

We require information from authors about some types of materials, experimental systems and methods used in many studies. Here, indicate whether each material, system or method listed is relevant to your study. If you are not sure if a list item applies to your research, read the appropriate section before selecting a response.

Materials & experimental systems

n/a	Involved in the study
☒	☐ Antibodies
☒	☐ Eukaryotic cell lines
☒	☐ Palaeontology and archaeology
☒	☐ Animals and other organisms
☒	☐ Clinical data
☒	☐ Dual use research of concern
☒	☐ Plants

Methods

n/a	Involved in the study
☒	☐ ChIP-seq
☒	☐ Flow cytometry
☒	☐ MRI-based neuroimaging

Plants

Seed stocks	*Report on the source of all seed stocks or other plant material used. If applicable, state the seed stock centre and catalogue number. If plant specimens were collected from the field, describe the collection location, date and sampling procedures.*
Novel plant genotypes	*Describe the methods by which all novel plant genotypes were produced. This includes those generated by transgenic approaches, gene editing, chemical/radiation-based mutagenesis and hybridization. For transgenic lines, describe the transformation method, the number of independent lines analyzed and the generation upon which experiments were performed. For gene-edited lines, describe the editor used, the endogenous sequence targeted for editing, the targeting guide RNA sequence (if applicable) and how the editor was applied.*
Authentication	*Describe any authentication procedures for each seed stock used or novel genotype generated. Describe any experiments used to assess the effect of a mutation and, where applicable, how potential secondary effects (e.g. second site T-DNA insertions, mosiacism, off-target gene editing) were examined.*
